# Regulation of the Human Telomerase Gene *TERT* by Telomere Position Effect—Over Long Distances (TPE-OLD): Implications for Aging and Cancer

**DOI:** 10.1371/journal.pbio.2000016

**Published:** 2016-12-15

**Authors:** Wanil Kim, Andrew T. Ludlow, Jaewon Min, Jerome D. Robin, Guido Stadler, Ilgen Mender, Tsung-Po Lai, Ning Zhang, Woodring E. Wright, Jerry W. Shay

**Affiliations:** 1 Department of Cell Biology, University of Texas Southwestern Medical Center, Dallas, Texas, United States of America; 2 Faculté de Médecine, Tour Pasteur 8éme Étage, Nice, France; 3 Berkeley Lights, Inc., Emeryville, California, United States of America; Lunenfeld-Tanenbaum Research Institute, Canada

## Abstract

Telomerase is expressed in early human development and then becomes silenced in most normal tissues. Because ~90% of primary human tumors express telomerase and generally maintain very short telomeres, telomerase is carefully regulated, particularly in large, long-lived mammals. In the current report, we provide substantial evidence for a new regulatory control mechanism of the rate limiting catalytic protein component of telomerase (h*TERT*) that is determined by the length of telomeres. We document that normal, young human cells with long telomeres have a repressed h*TERT* epigenetic status (chromatin and DNA methylation), but the epigenetic status is altered when telomeres become short. The change in epigenetic status correlates with altered expression of *TERT* and genes near to *TERT*, indicating a change in chromatin. Furthermore, we identified a chromosome 5p telomere loop to a region near *TERT* in human cells with long telomeres that is disengaged with increased cell divisions as telomeres progressively shorten. Finally, we provide support for a role of the TRF2 protein, and possibly TERRA, in the telomere looping maintenance mechanism through interactions with interstitial TTAGGG repeats. This provides new insights into how the changes in genome structure during replicative aging result in an increased susceptibility to age-related diseases and cancer prior to the initiation of a DNA damage signal.

## Author Summary

Telomerase is very tightly regulated in large, long-lived species such as humans. Telomerase is expressed during early human fetal development, turned off in most adult tissues, and then becomes reactivated in most human cancers. However, the exact mechanism(s) regulating these switches in expression are not fully known. We recently described a phenomenon in which genes near chromosome ends (telomeres) are regulated by telomere length-dependent loops (telomere position effect—over long distances; TPE-OLD). Interestingly, the *TERT* gene is only a megabase from the human chromosome 5p end. We observed that when telomeres are long, *TERT* gene expression is repressed and the 5p sub-telomeric region and the *TERT* locus are spatially co-localized. When telomeres are short, at least one of the *TERT* alleles is spatially separated from the telomere, developing more active histone marks and changes in DNA methylation in the *TERT* promoter region. These findings have implications for how cells turn off telomerase when telomeres are long during human fetal development and how cancer cells reactivate telomerase in cells that have short telomeres. These studies add to the growing support for the role of telomeres in regulating gene expression via TPE-OLD. Furthermore, telomere length may be one of the mechanisms of how cells time changes in physiology without initiating a DNA damage response.

## Introduction

All mammalian telomeres (the ends of linear chromosomes) are composed of large tracts of repeated 5ʹ-TTAGGG sequences. Telomeres are well-conserved DNA end structures from yeast to mammals, and it is believed that the primary role of telomeres, in combination with shelterin proteins, is to provide protection of the linear chromosome ends from being recognized as damaged or broken DNA [1] and to facilitate the completion of DNA replication each cell cycle. Telomeres prevent DNA end-joining, DNA recombination, and loss of essential genetic information during DNA replication. Telomeres are maintained by many essential genes, including the six-component shelterin (TRF1, TRF2, POT1, TIN2, RAP1, and TPP1) and the CST (CTC1-STN1-TEN1) complexes [1,2]. Impairment of these genes is closely associated with age-related clinical pathology and defects in germ cell and stem cell maintenance [3–5].

It is well established that hTERT, the catalytic core reverse transcriptase component, protein levels are rate-limiting for telomerase activity and telomere length homeostasis [6]. Human embryonic stem cells and transit amplifying adult progenitor stem-like cells express h*TERT* and have active/functional telomerase that can fully or partially maintain telomeres during the substantial number of cell divisions required in fetal development [7]. While telomerase is present from the blastocyst stage in early human development, at approximately 16–18 wk of gestation, telomerase activity is silenced in the vast majority of somatic cells [8]. The molecular mechanisms (i.e., transcriptional regulation, alternative splicing changes, epigenetic modifications, or other regulatory processes) that trigger the silencing of telomerase at specific times during human development remain uncertain. Irrespective, telomerase largely remains silent throughout adult life except for tumor development. In ~90% of human tumors, telomerase is upregulated or reactivated for the maintenance of telomeres during the numerous rounds of cell divisions required for the emergence of malignant and metastatic disease [9]. Thus, tight regulation of telomerase and progressive telomere shortening are thought to be an initial barrier to the early onset of cancer.

High resolution mapping of the three-dimensional chromatin interactome addresses many unanswered questions about the *cis*-regulatory long-range looping interactions important in gene regulation. The human genome is composed of continuous chromosome loops and TADs (topologically associating domains), forming gene territories [10,11]. Distal enhancers and/or insulators are believed to be responsible for the regulation of genes along the genome via chromatin folding. Recently, we demonstrated that telomeres also loop to specific loci to regulate gene expression, which we have termed TPE-OLD (telomere position effect—over long distance) [12–14]. In the examples characterized so far, genes close to telomeres are silenced in young cells (with long telomeres) and become expressed when telomeres are short. Importantly, re-elongation of cells with short telomeres by exogenous expression of the h*TERT* gene (active telomerase) results in expression patterns that mirror the expression of these genes in cells with long telomeres [12–15]. As we have observed genes between the TPE-OLD regulated genes that are not regulated by TPE-OLD, this mechanism is clearly distinct from classic TPE, which regulates genes proportional to the proximity to the telomeric repeats [15]. In the present study, we show that the expression of the h*TERT* gene itself is also regulated by TPE-OLD. The ability to regulate genes by telomere length without induction of a DNA damage signal from a single or a few critically short telomeres has potential explanatory value for what regulates the maximum length of human telomeres during fetal development and ways to regulate major age-associated transitions as well as to activate or repress genes as part of normal aging without requiring a DNA damage signal.

## Results

### Conserved *TERT* Loci in Higher Primates

**Fig 1:**
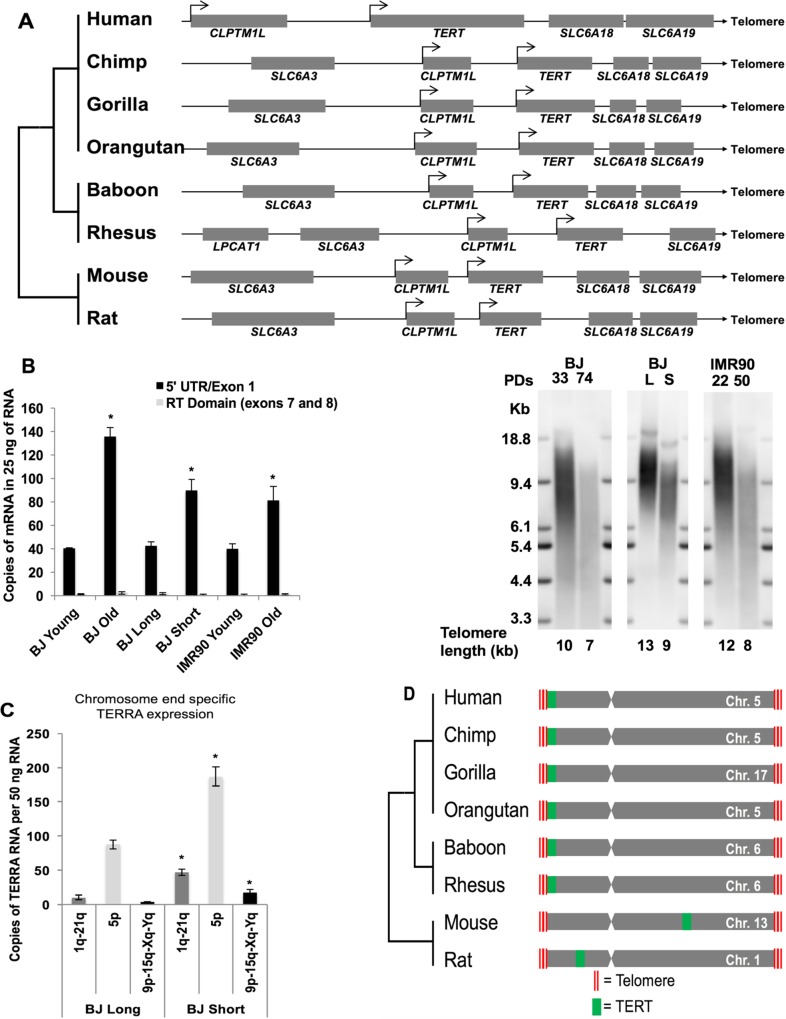
Conserved *TERT* loci in higher primates. (**A**) Conserved synteny map of *TERT* and *CLPTM1L* loci in mammals. Transcription orientation of *TERT* and location of telomere are shown in figure. (**B**) ddPCR analyses of the h*TERT* locus. mRNA expression in BJ cell lines at young population doublings (PD34) and old (PD74), BJ cell clones containing different telomere lengths, and IMR90 young (PD22) and old (PD50) were analyzed. RNA (1000ng) was reverse-transcribed, diluted, and 5ʹ hydrolysis probes (Roche UPL) were used to assess the number of mRNA molecules per reaction. Mean telomere length was analyzed by TRF in each of the analyzed cell lines. (**C**) Chromosome end-specific TERRA expression analysis was performed on BJ cell clones with long and short telomeres (same RNA as used above for *TERT* analysis). (**D**) Higher primates also retain the location of the *TERT* gene at the end of their chromosomes. Each bar represents an individual chromosome retaining the *TERT* locus. Location of the *TERT* gene is marked by green on the chromosome. Red bar represents location of telomeres. * = *p* < 0.05. kb = kilobases. Data are presented as means and standard errors of biological replicates and technical triplicates. Data associated with this figure can be found in the supplemental data file (S1 Data).

Long-ranged genomic interactions between telomeres and distal loci may play important roles in the regulation of gene expression, a phenomenon that we previously referred to as TPE-OLD [12,13]. Through previous microarray analyses [12], we identified the human *CLPTM1L* (cleft lip and palate-associated transmembrane protein 1-like) gene that is ~1.3 mega bases apart from the chromosome 5p telomere as a putative TPE-OLD candidate gene. *CLPTM1L* is frequently upregulated in cancer cells [16] and shows preserved colocalization with the *TERT* locus for a shared synteny in many species (**Fig 1A**). We analyzed mRNA expression of the genes at this locus, including *CLPTM1L* and h*TERT*, in BJ human fibroblast clones with long and short telomeres, to determine if the expression of this locus is regulated by TPE-OLD. *CLPTM1L* was expressed in normal young passaged cells but showed increased gene expression with progressive telomere shortening (**S1A Fig**). Historically, it is generally believed that h*TERT* is not actively transcribed in normal telomerase silent cells; however, expression of h*TERT* splice variants does occur [17]. The reason for this misconception is that most investigators use primer pairs designed to measure transcripts containing only the RT domain of *TERT* (exons 5–10), while exons outside of the RT domain are not measured (i.e., exons 1–4 and 11–16). It is now known that h*TERT* transcripts can be detected in a variety of telomerase-negative cells and tissues, but the mRNA produced is not full-length mRNA capable of producing active telomerase [17]. To test if replicative age or telomere length influenced h*TERT* expression, we measured h*TERT* gene expression using a primer pair targeting the 5ʹUTR to exon 1 of h*TERT*. We observed that h*TERT* is expressed at higher levels in two human fibroblast strains with short telomeres compared to the same cells with long telomeres (**Fig 1B**, **S1 Fig**). As previously described, we did not detect any transcripts that contain the RT domain of h*TERT* **(Fig 1B**); thus, transcripts that could code for active telomerase were not observed. We also analyzed protein expression of *CLPTM1L* (**S1B Fig**) and observed that the expression of CLPTM1L protein significantly increased during progressive telomere shortening, but the expression was greatly decreased when we re-introduced h*TERT* in old BJ cells and re-elongated telomeres (**S1B Fig**). We also examined mRNA expression of genes located between the 5p telomere and the *hTERT-CLPTM1L* locus (**S1A Fig**) in young and old BJ cells. The expression of the intermediate genes on chromosome 5p showed no significant increase in BJ cells with short telomeres (**S1A Fig**). We explored if telomere repeat containing RNA, TERRA, was also altered and potentially important in TPE-OLD. Consistent with previous reports [18,19] we observed an increase in TERRA expression from three subsets of chromosomes (1q-21q, 5p, and 9p-15q-Xq-Yq; **Fig 1C**) when telomeres were short compared to long. The TERRA data support our observations that the chromatin environment surrounding chromosome 5p and h*TERT* change when telomeres are short. Overall, this implies that the h*TERT* locus may be influenced by the length of telomeres through long-ranged chromatin interactions.

Perhaps not surprising, but potentially significant, is that the location of the *TERT* gene is also evolutionarily conserved (**Fig 1D**). *TERT* genes are located at the very end of their chromosomes, near the telomere, in higher primates including humans and most other large long-lived mammals. However, the location of the *TERT* gene in rodents and many other smaller shorter-lived mammals is non-telomeric. The local genome structure around the *TERT* locus in rodents is quite different from primates, implying they may have developed different strategies to regulate telomerase expression [20,21]. Based on these observations, we decided to test if there is a functional role for *TERT* being localized at the end of human chromosome 5p. As the distance between the h*TERT* locus and the telomere is only ~1.3 mega bases, we postulated that h*TERT* might also be regulated in part by a long-ranged telomere looping mechanism in human cells.

### Three-Dimensional Interactions between the h*TERT* Locus and the Sub-telomeric 5p Region by Telomere Length

**Fig 2:**
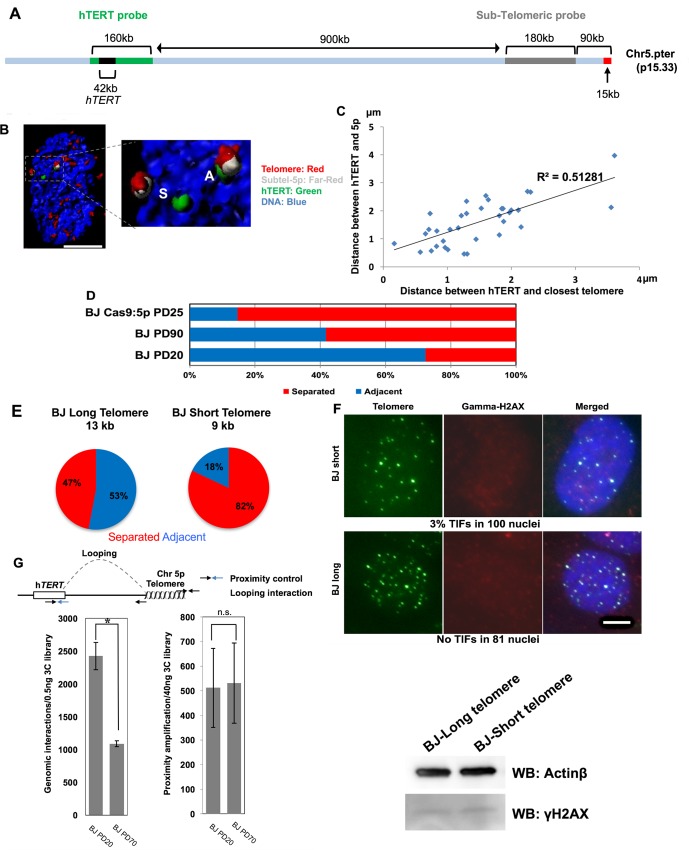
Three-dimensional interactions between the h*TERT* locus and the sub-telomeric 5p region by telomere length. (**A**) Schematic map of the human 5p chromosome containing the h*TERT* locus and design of probes. The sub-telomeric 5p probe (far, red, grey color) stains the end of chromosome 5p that is located 75 Kb from the 5p telomeric repeats (red color). The h*TERT* probe (green) stains a specific genomic region containing the h*TERT* gene, which is 1.1 Mb from the 5p telomere. (**B**) BJ human fibroblasts at PD60 was stained with indicated fluorescent probes against the h*TERT* locus, sub-telomeric region 5p, and the telomere. A representative deconvolved image was selected. Scale bar represents 5 uM (**C**) Graph shows the distance between the h*TERT* locus and the closest telomere depends on the distance between the h*TERT* locus and the sub-telomeric 5p. (**D**) Percentage of adjacent allele (A) pairs versus separated allele (S) pairs was determined by 3D-FISH in normal BJ cells. BJ fibroblasts at different PDs were analyzed as indicated in the Materials and Methods. Cas9-mediated transient perturbation at the sub-telomeric 5p region was performed in BJ cells at PD25. (**E**) Percentage of adjacent allele pairs (A) versus separated allele pairs (S) was determined by 3D-FISH in BJ fibroblasts. BJ clones were transfected with a floxable h*TERT*, followed by excision with Cre recombinase at different time points. Two clones with different lengths of telomeres were analyzed at the same number of population doublings in culture. Indicated telomere lengths were measured by TRF (terminal restriction fragment) Southern blot shown in Fig 1C. (**F**) Telomere-DNA damage induced foci (TIF) analysis performed in BJ long (13 kb) and short cells (9 kb) (TRF shown in Fig 1C for matched cells). Cells were stained with telomere PNA and gamma-H2AX antibody. At least 81 nuclei were quantified. Scale bar is 5 μM. Western blotting shows lack of induction of γ-H2AX in BJ cells with different telomere lengths. β-Actin was used as a loading control. (**G**) 3C analysis shows distal genomic interactions between the 5p telomere and h*TERT*. 3C libraries were generated from BJ cells at PD20 and PD70, followed by ddPCR amplification of genomic interactions between indicated regions. Proximity control amplification of 3C libraries from BJ cells at PD20 and PD70 was performed with ddPCR. *t* test revealed a significant effect. *p < 0.05, *n*.*s*. = no significant. Data associated with this figure can be found in the supplemental data file (S1 Data).

We designed two specific BAC probes to visualize the h*TERT* locus and the sub-telomeric 5p region for three-dimensional fluorescence in situ hybridization (3D-FISH) (**Fig 2A**). We measured the distance between the h*TERT* locus and the sub-telomeric 5p region, and the pairs of alleles were divided into adjacent to (A) or separated (S) by the three-dimensional location (**S2A and S2B Fig**). We first stained the sub-telomeric BAC region, the h*TERT* locus and telomeres in old BJ cells, with short telomeres (**Fig 2B**). The telomere staining was detected at the h*TERT* locus with sub-telomere 5p in the adjacent allele pair. However, we observed at least one h*TERT* allele that was spatially separated from the sub-telomere 5p probe in old BJ cells without telomere staining. We measured the distance between the h*TERT* locus and the closest telomere (**Fig 2C**). The results showed that the h*TERT* locus colocalized with the telomere when it is adjacent to the sub-telomeric 5p region (**Fig 2B and 2C**). This implies that the telomere is likely to be adjacent to the h*TERT* locus for potential long-ranged looping interactions.

We next tested if telomere looping close to the h*TERT* locus changes when telomeres became short. We measured and compared the distance between the h*TERT* locus and sub-telomeric 5p in young BJ fibroblasts at 20 population doublings (PDs) with long telomeres versus old BJ fibroblast at PD90 with short telomeres (**Fig 2D**). More than 70% of allele pairs were adjacent in BJ cells at PD20, implying that the telomeric heterochromatin might affect the expression of the h*TERT* locus in young BJ fibroblasts. BJ cells are telomerase-negative, but non-catalytic alternatively spliced variants are expressed, as shown in Fig 1 and as previously described [17]. This might explain why a small proportion of alleles is separated from the telomere in telomerase-negative young BJ cells with long telomeres, based on the assumption that the looping interactions suppress transcription. In old BJ cells at PD90, we found that the percentage of adjacent allele pairs was significantly reduced. Almost 60% of alleles were separated in the old cells with short telomeres, indicating that there is at least one h*TERT* locus spatially separated from the telomere in each cell. Importantly, we confirmed these 5p/TERT looping interactions in a second fibroblast cell strain, IMR90 (**S2C and S2D Fig**). We measured the number of separated and adjacent alleles in IMR90 cells young (PD 22) and old (PD 52) and show a shift from the majority of alleles being adjacent (76%) in young cells compared to the majority of alleles being separated (88%) in old cells. The looping data and the expression of h*TERT* are consistent. We suggest that old cells (with short telomeres) lose one control mechanism in regulating the h*TERT* locus (i.e., telomere chromatin looping) that helps repress the expression of h*TERT*. However, while we observed increased transcription of exon 1 of h*TERT*, there must be additional mechanisms preventing the inclusion of exons critical to produce active telomerase. There is substantial evidence that alternative splicing of h*TERT* may also have a major role in suppressing the production of active telomerase in old cells [22–24]. Furthermore, we performed 3D-FISH analysis in transformed SW26 and SW39 cells. SW cells are SV40 antigen expressing clones of IMR90 cells that have spontaneously immortalized using either telomerase (SW39) or an alternative lengthening of telomeres (ALT; SW26) mechanism to maintain telomeres (**S2E Fig**). In both cell lines, the majority of the alleles were separated (SW39 = 72%; SW26 = 66%), indicating that short telomeres due to replicative aging are likely responsible for the change in chromatin conformation and that a secondary change occurs to cause the production of full-length TERT or engage ALT.

It has been suggested that h*TERT* shows mono-allelic expression in cancer, which is sufficient to preserve constant telomere length [25,26]. Our results support this assumption, as we observed that, on average, only one h*TERT* allele was generally in the open configuration during in vitro aging well before the onset of cancer. As controls for global conformational changes at chromosome 5p, we performed two additional FISH experiments. In the first experiment, we stained intermediate genomic region between the h*TERT* locus and the 5p telomere (**S3A–S3C Fig**). In addition, we also stained cells for two loci located 25.5 MB and 30.6 MB away from h*TERT* (**S3D–S3F Fig**). There were no changes in distances between the control loci in young and old cells, demonstrating that the conformation change occurs at the specific genomic region encompassing h*TERT* during in vitro aging, and this change is not due to classic TPE.

To determine if we could artificially shorten telomeres and recapitulate the aging phenotype, we utilized CRISPR/Cas9 (clustered regularly interspaced short palindromic repeat-associated 9) to remove a large portion of the telomere and subtelomere region from chromosome 5p. This experiment allows testing the role of chromosome 5p’s telomere in regulating the looping observed in cells with short and long telomeres. As illustrated in **Fig 2D**, we also infected young BJ cells with a lentivirus expressing Cas9 protein and single guide-RNA targeting the sub-telomeric region of 5p to specifically disturb telomeres at chromosome 5p for a short period of time [27]. We also added an NHEJ inhibitor, SCR7, simultaneously during the infection to suppress repair of the double strand breaks induced by the Cas9 protein [28]. The targeted cells showed an unstable end structure of chromosome 5p (**S5 Fig**), and the specific disturbance of the 5p telomere significantly diminished telomere looping at the end of the chromosome 5p.

We further examined if the proposed mechanism was present in BJ cell clones in which both young and old cells were passaged the same amount of time in culture. This approach was necessary to eliminate the possibility that young and old cells that were in culture for vastly differing times could introduce artifacts. To accomplish this, we expressed a floxable h*TERT* in BJ clones, followed by excision at different time points in order to make isogenic cells with different length of telomeres but passaged similar times in cell culture [12,29]. Telomere length of the early-excision clone was 9 kb, and this was extended up to 13 kb in the late-excision clone. The telomere length (terminal restriction fragment [TRF]) results are presented in Figure 1B. Population doublings were evenly matched between clones (to avoid confounding effects of passage of time in culture), and we also analyzed telomere looping. Similar to our observations in normally passaged BJ cells, the isogenic clones also showed decreased levels of telomere looping with telomere shortening (**Fig 2E**). Importantly, there were only background levels of DNA damage signaling during telomere shortening (**Fig 2F**) indicating that the change in genome structure occurred before initiation of DNA damage responses from critically short telomeres. To ensure that our staining protocol was robust, we induced DNA damage (double strand breaks) by treating long and short telomere BJ cells with zeocin and assaying for DNA damage (**S2F Fig**). These data can be interpreted to indicate that our staining protocol is robust and that we are analyzing cells before telomere-DNA damage induced foci are present or significant DNA damage occurs in the cells.

We next performed droplet digital 3C (chromatin conformation capture) to detect the genomic interactions between the 5p telomere and the h*TERT* locus in young and old BJ cells (**Fig 2G, left side**). The results showed that the h*TERT* locus has specific genomic interactions with the 5p telomere, and the interaction was reduced during *in vitro* aging and telomere shortening. A proximity control primer which is 10kb away from the fixed primer at the h*TERT* locus was selected for normalization of 3C results (**Fig 2G, right side**). Taken together, telomere looping exists between the h*TERT* locus and the sub-telomeric 5p in normal human cells, and this looping is greatly reduced by gradual telomere shortening.

### Telomere Looping Determines Permissiveness of the h*TERT* Locus

**Fig 3:**
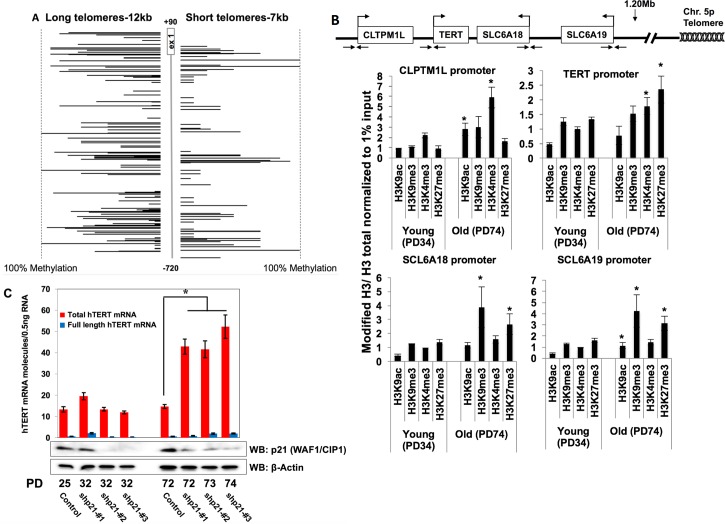
Chromatin looping and epigenetic modifications (i.e., histone modifications and DNA methylation) determine permissiveness of the h*TERT* locus. (**A**) Bisulfite DNA methylation sequencing analysis of the h*TERT* proximal promoter region from -720bp to +90bp. Genomic DNA of BJ clones with different lengths of telomeres was modified and PCR-amplified. Each amplicon was TA-cloned for bacterial amplification and sequenced. Percentage of CpG methylation of the hTERT promoter is indicated in two BJ cell clones with different lengths of telomeres. (**B**) Illustration of genomic locus containing *TERT*. Black arrows indicate approximate location of primers in the promoters of the indicated genes. Chromatin immunoprecipitation was performed with BJ cells at PD34 and PD74. Six antibodies against H3K4me3, H3K9ac, H3K9me3, H3K27me3, H3 total, and IgG were used to pull down chromatin extracts, and the promoter regions of h*TERT*, *SLC6A18*, *SLC6A19 and CLPTM1L* were analyzed by ddPCR. Data are presented as means and standard errors of biological and technical duplicates. Student’s paired *t* tests comparing young and old determined significance (* = *p* < 0.05). (**C**) BJ cells at different PDs were infected with shRNAs against p21 (*CDKN1A*) and selected using puromycin to generate stable knockdown clones. h*TERT* mRNA expression was analyzed by ddPCR in sh-p21 cells and controls. The number of full-length and total h*TERT* mRNAs was assessed by amplifying the h*TERT* exon 7/8 junction and exon 15/16 junction, respectively. Knockdown efficiency of p21 was determined by western blotting. β-actin was used as a loading control. ANOVA revealed a significant effect. *p < 0.001. Data associated with this figure can be found in the supplemental data file (S1 Data).

It has been shown that *cis*-elements upstream of the h*TERT* locus may play important roles in the tight regulation of human telomerase [30]. Thus, we decided to test if telomere looping could affect the epigenetic status of the h*TERT* proximal promoter region. We first analyzed DNA methylation of the region from -720bp to +90bp of the *hTERT* promoter in isogenic BJ cells with different lengths of telomeres but similar times in cell culture (**Fig 3A**). The relationship between DNA methylation and transcription in the h*TERT* promoter remains controversial in normal and cancer cells [31,32], but the transcription start site of h*TERT* retains little or no methylation in telomerase-active cancer cells for active transcription [33]. We found that the level of DNA methylation is significantly higher in BJ cells with long telomeres at several regions associated with h*TERT* and the h*TERT* region in comparison to cells with shorter telomeres. The largest differences were observed at -580bp, -250bp, -30bp, and +20bp of the h*TERT* promoter, including the E-box motif (a putative Myc binding sequence). It has also been reported that the proximal region of the h*TERT* promoter, including exon 1 and 2, regulates the activity of the h*TERT* promoter and that the methylation of this region is responsible for binding of several proteins [34,35]. Therefore, our results can be interpreted to indicate that telomere length-associated changes in methylation levels of the h*TERT* proximal promoter might affect transcriptional regulation of this locus. We next analyzed active and inactive histone marks on the h*TERT* proximal promoter using chromatin immunoprecipitation combined with droplet digital polymerase chain reaction (ChIP-ddPCR; [12]) (**Fig 3B**). We measured two histone marks associated with active chromatin H3K4 trimethylation (H3K4me3) and H3K9 acetylation (H3K4ac) and two histone marks associated with repressed chromatin H3K27 trimethylation (H3K27me3) and H3K9 trimethylation (H3K9me3), which have key roles in regulating gene expression [36]. We observed an increase in both H3K4me3 and H3K9ac across the *TERT* promoter in aged cells with short telomeres (**Fig 3B**). We also observed an increase in the repressive histone mark H3K27me3, but did not observe any significant differences in young or old BJ cells for the repressive histone mark H3K9me3. Collectively, this shows that the chromatin status of the h*TERT* promoter in old BJ cells with short telomeres is different and may be more transcriptionally permissive compared to young BJ cells with long telomeres. These data correlate well with the increased h*TERT* transcription we observed in cells with short telomeres. Furthermore, we analyzed chromatin at the promoters of three genes surrounding *TERT* that could also be affected by the altered chromatin environment with aging. We analyzed the proximal promoter regions of *CLPTM1L*, *SCL6A18*, and *SCL6A19* for the same histone marks described above in the same cells and preparations used for *TERT* ChIP. At the *CLPTM1L* promoter we observed significant increases in histone marks indicating active transcription (**Fig 3B**). These data correlate well with an increase in *CLPTM1L* transcripts and protein levels (**S1 Fig**). We also observed significant changes in the chromatin surrounding the solute/amino acid transporter genes (*SCL6A18* and *SCL6A19*), even though these genes are not expressed above basal/background levels in old/short telomere BJ cells. Specifically, we observed that both the repressive histone marks were increased in old cells (short telomeres) compared to young cells (long telomere). However, there was an increase in the activation marks as well. This indicates an intricate balance between chromatin modifications, methylation status, telomere length, and the expression of tissue-specific transcription and splicing factors that dictates the activation or repression of genes with replicative aging (telomere shortening—TPE-OLD).

While we demonstrated that telomere shortening induced conformation changes between the h*TERT* locus and the sub-telomeric 5p resulting in up regulation of exon 1, presumably containing spliced h*TERT* transcripts in normal BJ cells (see **Fig 1B**), it did not result in full-length telomerase activity competent transcripts. Thus, we suggest that telomere shortening may render the h*TERT* locus more permissive and under oncogenic stress may lead to the production of full-length h*TERT* mRNA transcripts that could in turn produce telomerase activity. To test this, we simulated a step in spontaneous cancer transformation by knocking down p21 (*CDKN1A*) and analyzing mRNA expression level of h*TERT* (**Fig 3C**). The knockdown of p21 was previously shown to de-repress h*TERT* expression [37]. Thus, we tested if the knockdown of p21 would increase the expression of h*TERT* mRNAs and result in the inclusion of exons 7/8 in the short-telomere old BJ cells but not in the young BJ cells with long telomeres. We measured the expression level of h*TERT* transcripts in young and old BJ cells with and without p21 stable knockdown; mRNA containing exons 7/8 (exons coding for critical residues in the reverse transcriptase domain of *TERT*) and exon 15/16 (most splice variants of h*TERT* contain exons 15 and 16), responsible for putative active h*TERT* and total h*TERT* variants respectively. Both the active and the total h*TERT* transcript variants significantly increased with the knockdown of p21 in old BJ but not in young BJ cells; however, we did not detect telomerase activity (**S6 Fig**). While we observed an increased portion of transcripts that contain exons 7/8 of the TERT RT domain, other critical regions such as exon 2 may be spliced out [38]. Further work into the regulation of h*TERT* splicing is necessary to more fully understand the complex regulatory network surrounding h*TERT* and why the majority of transcripts are inactive splice variants as opposed to full length. While this result does not prove a causal role during cancer development, this series of experiments does demonstrate that telomere shortening in cells that bypass replicative senescence leads to the h*TERT* locus entering into a more permissive state (e.g., increased h*TERT* mRNA expression) in the presence of oncogenic stresses, consistent with the disengagement of telomere looping.

### TRF2 as a Mediator of Telomere Looping between the h*TERT* and Sub-telomeric 5p in Cells with Long Telomeres

**Fig 4:**
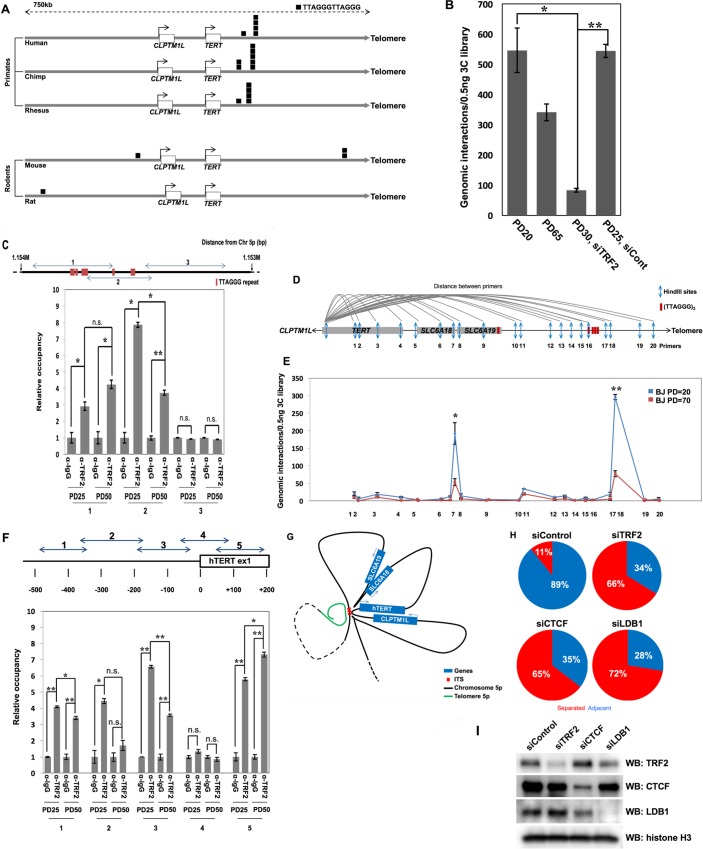
TRF2 as a mediator of telomere looping between the h*TERT* and sub-telomeric 5p in cells with long telomeres. (**A**) The number of (TTAGGG)_2_ repeats was analyzed on a small series of mammalian genomes. The *TERT* locus flanked by 350kb was examined. The (TTAGGG)_2_ repeats are depicted as a single black box. Multiple (TTAGGG)_2_ repeats at a locus are depicted by stacked black boxes. Transcription orientation of *TERT* and location of telomere are shown in figure. (**B**) 3C analyses show distal genomic interactions between the 5p telomere and h*TERT*. 3C libraries were generated from BJ cells treated with siRNA against TRF2, followed by ddPCR amplification to determine genomic interactions. *t* test revealed a significant effect. *p < 0.05, **p < 0.01 (**C**) ChIP shows enrichment of TRF2 protein on h*TERT*-ITS. The 1.153 Mbp to 1.154 Mbp region from the end of the chromosome 5p was examined. Chromatin extracts from BJ cell at PD25 and PD50 were prepared and pulled down with an antibody against TRF2. TTAGGG repeats are depicted as a red box. (**D**) A schematic map of the human chromosome 5 at the 1,153,167 bp to 1,353,167 bp region from the p arm terminus. Arrows indicate location of HindIII restriction enzyme sites. Red boxes indicate location of TTAGGGTTAGGG repeats along the genome. (**E**) 3C analysis shows distal genomic interactions between 5`end of h*TERT* locus and each HindIII site. 3C libraries were generated from BJ cells at PD25 and PD70, followed by ddPCR amplification to determine genomic interactions. *t* test revealed a significant effect. *p < 0.05, **p < 0.01. (**F**) ChIP shows enrichment of TRF2 protein on the h*TERT* promoter. Chromatin extracts from BJ cell at PD25 and PD50 were prepared and pulled down with an antibody against TRF2. The h*TERT* promoter region from -500bp to +200bp was analyzed by real-time-qPCR. *t* test revealed a significant effect. *p < 0.05, **p < 0.05, n.s. = no significance. (**G**) Model of genomic folding at h*TERT* locus based on 3C analyses. Blue boxes indicate the location of genes on chromosome 5p. Red boxes indicate the location of h*TERT*-ITS. Blue and red dashes indicate chromosome 5p and telomere of chromosome 5p, respectively. (**H**) BJ cells at PD 17 were transfected in individual experiments with a siRNA against TRF2, CTCF, and LDB1. Three days after transfection, cells were fixed for 3D-FISH analysis. Percentage of adjacent allele (A) pairs versus separated allele (S) pairs was determined by 3D-FISH. (**I**) Western blotting analysis shows knockdown efficiency of the siRNA against TRF2, CTCF, and LDB1. Histone H3 was used as a loading control. Data associated with this figure can be found in the supplemental data file (S1 Data).

Characterization of *cis*- or *trans*-acting factors responsible for telomere looping will be important to understand this novel mechanism for telomerase regulation. A recent report showed that TRF2 (telomeric repeat-binding factor 2) protein is essential for the functional organization of chromosome ends, including human fibroblasts [39,40]. There is also mounting evidence for off-telomere functions of the shelterin components [41]. While a recent whole genome sequencing study found 2,920 interstitial TTAGGG repeats throughout the human genome [39], we also found frequent internal (interstitial) telomeric sequences (ITS) near the *TERT* locus in higher primates but not in rodent cells (**Fig 4A**). Thus, we first checked for a putative role of TRF2 in telomere looping in BJ cells as a candidate approach. We knocked down TRF2 by siRNA and performed 3C to directly assess the genomic interactions between the telomere and the h*TERT* locus (**Fig 4B**). The knockdown of TRF2 significantly reduced the genomic interactions between the telomere and the h*TERT* locus in young PD30 BJ cells, implying TRF2 may have a role in telomere looping interaction on h*TERT* locus.

As shown in **Fig 4A,** a region 100 kb downstream of the h*TERT* (Chr5: 1,154,047–1,154,347) contains a series of internal telomeric sequences that may recruit TRF2 shelterin protein (hereafter termed h*TERT*-ITS). Thus, we reasoned that this region would be a putative binding site for TRF2 and may be responsible for the telomere looping interaction between the telomere and the h*TERT* locus in cells with long but not short telomeres. ChIP-qPCR analysis showed that the TRF2 protein associates with the h*TERT*-ITS region in young and old BJ cells as proposed (**Fig 4C**). We next performed 3C to further clarify that h*TERT*-ITS interact with the h*TERT* promoter by genome folding to affect transcriptional permissiveness as shown in **Figs 1** and **3**. Within 200kb, we found more than 20 HindIII restriction enzyme sites were in the h*TERT*/*CLPTM1L* locus (**Fig 4D**). Droplet digital PCR (ddPCR)-mediated amplification showed specific interactions between the 5ʹ end of h*TERT* and the h*TERT*-ITS (**Fig 4E**). Moreover, the interaction was weakened in old BJ cells, implying there might be a transition from a more repressive state to a more active state of this TAD location during in vitro aging, consistent with the increased h*TERT* mRNA, altered methylation, and chromatin. This result also shows that there is an additional genome folding between the h*TERT* locus and the h*TERT*-ITS at an intermediate region between the *SLC6A18* and *SLC6A19* loci. The h*TERT* promoter is not close to the h*TERT*-ITS on a linear genome map, but the unique genome folding at this region potentially positions the h*TERT* promoter close to the ITS, followed by putative TRF2-mediated telomere recruitment to the h*TERT* promoter only in cells with long telomeres.

In **Fig 4F**, we demonstrate that TRF2 protein is also enriched in the h*TERT* promoter region using ChIP-qPCR approaches. While TRF2 protein was enriched at proximal regions on the h*TERT* promoter, the interaction was significantly decreased in old BJ cells at the genomic regions containing -350bp to -50bp of the h*TERT* promoter. This shows TRF2 protein can occupy the h*TERT* promoter region, but the interaction is weakened during in vitro aging and telomere shortening. Together, we interpret these experiments to indicate that TRF2, and perhaps upregulated TERRA, may have at least a partial mechanistic role in telomere looping at the h*TERT* locus through interaction with the conserved interstitial telomeric repeats.

Because we have shown the interaction between the 5p telomere and the h*TERT* locus, we modeled one possibility for the detailed local genome structure of this locus (**Fig 4G**). In this model, the h*TERT* promoter is close to the h*TERT*-ITS by genome folding in young cells with long telomeres. In addition, this model shows that TRF2 protein is recruited to h*TERT* locus and h*TERT*-ITS, which makes this interaction potentially dependent on telomere length. In summary, the h*TERT* promoter has specific interactions with the h*TERT*-ITS through gene looping, which may also recruit telomere length-dependent looping (TPE-OLD) mechanisms through TRF2 protein.

We next performed 3D-FISH to visualize the genomic structure changes between the h*TERT* locus and the sub-telomeric 5p (**Fig 4H**). Control PD17 BJ cells showed that 89% of the h*TERT* and sub-telomeric 5p allele pairs were adjacent, but knockdown of TRF2 reduced this down to 34%. We also knocked down CTCF (CCCTC-binding factor) and LDB1 (LIM domain-binding protein 1), which are proposed to be essential proteins in global gene looping maintenance [42,43]. CTCF and LDB1 knockdown also significantly reduced the adjacent allele pairs, implying that the general gene looping mechanisms may also be involved in telomere looping. Western blotting was also performed to show knockdown efficiency (**Fig 4I**). Taken together, TRF2, part of the shelterin complex, may be mechanistically involved in the establishment of telomere looping near the h*TERT* locus through ITS together with general chromosome looping mechanisms.

### Telomere Length Affects Expression of h*TERT* in Telomerase-Positive Cancer Cells

**Fig 5:**
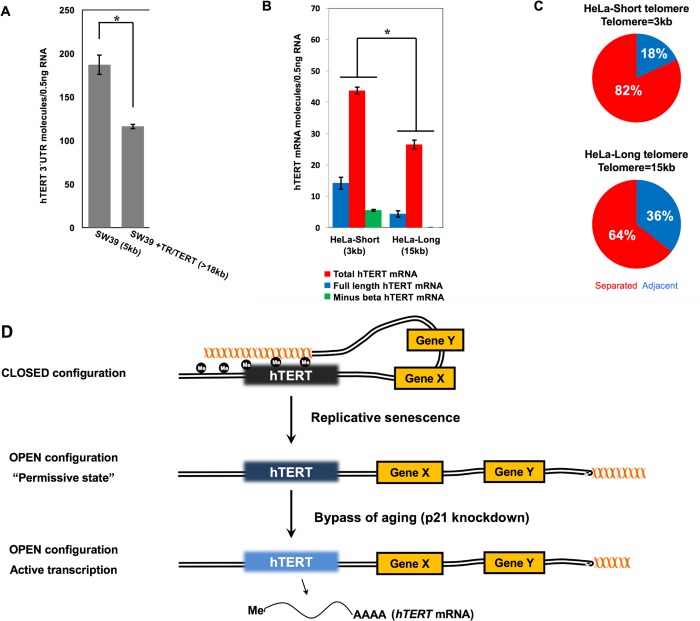
Telomere length affects expression of h*TERT* in telomerase-positive cancer cells. (**A**) SW39 cells were infected with viruses for ectopic expression of h*TERT* and h*TR* (h*TERC*). RNA was purified, and the 3ʹUTR was amplified to analyze endogenous level of h*TERT* mRNA. ddPCR was performed to assess relative expression levels. *t* test revealed a significant effect. *p < 0.005 (**B**) HeLa cells were cloned and infected with a floxable h*TERT*. After treatment of adeno-cre recombinase at different time points, HeLa clones with long and short telomeres at same number of population doublings in cell culture were established. The number of full-length, total, and minus beta h*TERT* mRNAs were assessed by amplifying exon7/8 junction, exon 15/16 junction, and the exon 6/9 junction respectively. Telomere lengths are determined by TRF and indicated under the graph. *t* test revealed a significant effect between splice variants. *p < 0.001 (**C**) Percentage of adjacent allele (A) pairs versus separated allele (S) pairs was determined by 3D-FISH in HeLa clones. HeLa clones with long and short telomeres at the same PD in culture were analyzed. (**D**) Simplified model of how TPE-OLD regulates h*TERT* expression in human cells during aging and cancer progression. See text for details. Data associated with this figure can be found in the supplemental data file (S1 Data).

In almost all primary human cancers, telomere length is very short compared to adjacent normal tissues [44]. It is likely that short telomeres, in combination with oncogenic alterations, result in the h*TERT* gene becoming more permissive for protein expression and enzyme activity. Thus, we next investigated how telomere length affects h*TERT* expression in telomerase-active cancer cells. We first infected h*TERT* and h*TR* (h*TERC)* into the SW39 cell line (SV40 immortalized human telomerase expressing fibroblasts) and analyzed mRNA expression of the endogenous h*TERT* by examining the 3ʹ untranslated region. We observed that the extended telomere length reduced endogenous expression of h*TERT* mRNA in qPCR analysis (**Fig 5A**) implying TPE-OLD remains engaged at least in this tumor cell line.

We further established isogenic HeLa cell clones with different telomere lengths by excising a floxable h*TERT* cDNA at different time points. We examined expression of splice variants of h*TERT* mRNA containing total, full-length (indicative of telomerase activity), and minus beta alternative spliced forms through ddPCR analysis (**Fig 5B**). All three splice variants showed significantly decreased expression in the long-telomere HeLa clone. We also performed 3D-FISH to analyze changes in genomic structure between the h*TERT* locus and the sub-telomeric 5p after the extension of telomeres in HeLa cells (**Fig 5C**). The long-telomere HeLa clone showed a higher percentage of adjacent allele pairs compared with the short-telomere HeLa clone. This indicates that the expression of h*TERT* may also be influenced by the length of telomeres through TPE-OLD in telomerase-positive cancer cells.

## Discussion

The local genome structure around the h*TERT* locus may be important for the tight regulation of human telomerase. For example, introduction of proximal *cis*-elements of the h*TERT* promoter sufficiently inhibits the activity of the *TERT* promoter [45]. In addition, chemicals perturbing chromatin structure, including trichostatin A and 5-aza-2ʹ-deoxycytidine, induce changes in h*TERT* expression [46]. Moreover, chromosomal translocation and gene duplication of the h*TERT* locus can occur as part of the immortalization process in primary cultured cells [47,48]. Here, we reasoned that the h*TERT* locus might recruit telomeric heterochromatin to regulate its own gene expression, especially in large, long-lived mammals where tumor suppression mechanisms are perhaps more important. We showed that telomere looping exists in long-telomere young fibroblasts and that telomere looping was reduced by in vitro aging. This is one possible explanation for why higher primates preserved the location of the *TERT* gene at the end of one of their chromosomes. We speculate that, in addition to other conserved tumor suppressor mechanisms, higher primates also developed a mechanism to suppress the undesired expression of *TERT*. For example, it is well established that during human fetal development, full-length telomerase transcription is repressed and correlates with increases in nonfunctional alternative splicing changes in h*TERT* [8]. Thus, during early human development, when telomerase is active, telomeres elongate. Our current results are consistent with the idea that longer telomeres can fold back on the *TERT* locus and repress or significantly reduce transcription. Our results also show that replicative senescence, while initially a tumor suppressor mechanism, may paradoxically impinge on the predisposition to cancer through telomerase transcriptional de-repression.

While still preliminary, the h*TERT* locus is arranged in a local chromatin domain that is regulated by telomere length and the interstitial telomere sequences in the vicinity of the h*TERT* locus. We showed that expression of the *CLPTM1L* gene (adjacent to h*TERT*) is also regulated by the length of telomeres and predicts transcriptional permissiveness of this locus. However, because h*TERT* re-activation is an extremely rare event, there may be additional levels of regulation. We propose that, upon telomere shortening, the h*TERT* region becomes permissive (as indicated by increased transcription of exon 1 containing RNAs), but this first step is not sufficient to support full-length h*TERT* transcripts at an adequate level to produce telomerase enzyme activity. We further propose that there is another biological role for telomere looping at this locus during development to repress telomerase when telomere length homoeostasis is reached (i.e., suggesting that having too-long telomeres may be detrimental).

Here, we demonstrated a novel epigenetic mechanism regulating h*TERT* expression during in vitro aging (**Fig 5D**). Cells with long telomeres at the end of chromosome 5p in young passaged cells form a chromatin loop in the region of the h*TERT* locus. Importantly, we demonstrated that the chromatin loop is disengaged in cells with short telomeres, leading to partial increased expression of h*TERT* mRNA during in vitro aging and in response to p21 knockdown; however, telomerase activity was not detected, and, alternatively, spliced variants were likely produced [17,22–24,38]. Finally, we demonstrated that, in old cells with short telomeres, re-introduction of h*TERT* and elongation of telomeres results in a re-engagement of TPE-OLD. We found that DNA methylation and histone modifications in the h*TERT* promoter region showed significant changes as cells developed shorter telomeres, and that TRF2 and, perhaps, TERRA, may have important roles in these age-dependent genomic changes. These observations offer a model and a partial explanation for how age-dependent changes in the genome structure affect the regulation of h*TERT* without initiating a DNA damage response from a critically shortened telomere.

## Materials and Methods

### Cell Culture

BJ, SW39, HeLa, HEK293FT, IMR90, and Phoenix A cells were maintained in a 4:1 ratio of Dulbecco’s modified Eagle’s medium to Medium 199 containing 10% of fetal bovine serum (Hyclone, Logan, UT, USA) under 5% CO_2_ in a humidified incubator. Retrovirus containing human *TERT* cDNA was infected into BJ cells and HeLa cells, followed by adenoviral infection for transient expression of Cre recombinase at different time points to produce cells with different lengths of telomeres that had been passaged in vitro for similar times [12,29]. Retrovirus was prepared by transfecting viral vectors into Phoenix A cells for 48 h. Medium containing virus was filtered through a 0.45 μm pore and provided to cells in the presence of 2 μg/ml polybrene. Lentivirus was prepared by transfecting viral vectors into HEK293FT cells with two packaging vectors (pMD2 and psPAX2) for 48 h. Medium containing virus was filtered through a 0.45 μm pore and cells exposed to lentivirus in the presence of 2 μg/ml polybrene. Selection for hygromycin was performed using 100 μg/ml and for puromycin using 1 μg/ml. CRISPR-Cas9 introduction for 5p genomic editing was performed by infecting cells with lentivirus carrying sgRNA target sequence of 5ʹ-GCCTCACTCCTTACGGAGTG-3ʹ.

### Three-Dimensional Fluorescence In Situ Hybridization (3D-FISH)

3D-FISH was performed as described previously [12]. 10^4^ BJ cells were seeded into 4-chambered slides. Cells on slides were fixed with 4% paraformaldehyde, followed by permeabilization with 0.1% Triton X-100 in PBS. Repeated liquid nitrogen freezing-thawing cycles were performed for further permeabilization with preservation of intact nuclear structure under 20% glycerol in PBS. After 5 d of incubation of with 50% formamide in 2X SSC, cells were stained with indicated probes at 37°C for overnight. Slides were washed with 0.1% SDS in 0.5X SSC at 70°C for 5 min, followed by 2 rounds of PBST (Phosphate-buffered saline with Triton X-100) washing for 10 min. Images were acquired using a LSM780 confocal microscope (Carl Zeiss, Jena, Germany), and analyzed by Imaris deconvolution software (Bitplane, Zurich, Switzerland). The proximity of allele pairs was determined visually and quantitated. At least 30 nuclei were counted for the statistical analyses. We used the following criteria for the analyses: adjacent ~0.5 μm space or less between probes, separated ~1.0 μm between probes or more. The length was determined by calculating the 3D distance between each center of deconvolved fluorescent spots. Probes were prepared using nick translation kits (Abbott Laboratories, Abbott Park, IL, USA) from each BAC following manufacturer’s instructions. BAC plasmids were purchased from CHORI (Children’s Hospital Oakland Research Institute, Oakland, CA, USA); RP11-990A6 for h*TERT* locus staining and RP11-44H14 for sub-telomeric region 5p staining. Quality of probes was assessed by metaphase spread analyses and PCR.

### Droplet Digital PCR (ddPCR) and TRAP (ddTRAP)

DdPCR and ddTRAP were performed as previously described [12,49]. Messenger RNA was prepared from RNeasy plus mini kit (Qiagen, Valencia, CA, USA) following the manufacturer’s instructions. 100 ng of RNA was reverse-transcribed from cDNA synthesis kit (Bio-Rad, Hercules, CA, USA) by following the manufacturer’s instructions. Ten percent of synthesized cDNA was used for the ddPCR reaction. For ddTRAP, harvested cells were lysed in NP40 lysis buffer (1mM Tris-Cl pH8.0, 1mM MgCl_2_, 1mM EDTA, 1% NP40, 0.25mM sodium deoxycholate, 10% glycerol, 0.15M NaCl, 0.05% 2-ME) for ddTRAP. Lysate was used for TS extension, and the extended products were analyzed with ddTRAP. Endogenous levels of 3ʹUTR were assessed with EvaGreen dye (Bio-Rad, Hercules, CA, USA). Probes were purchased from Roche (Basel, Switzerland), and the primer sequences are described below:

5ʹUTR-Exon 1 hTERT
5ʹ-AGCCCCTCCCCTTCCTTT
5ʹ-TGCGTCCCAGGGCACGCACACCAGGCACTG
Full-length hTERT (exon7/8)
5ʹ-GCGTAGGAAGACGTCGAAGA-3ʹ
5ʹ-ACAGTTCGTGGCTCACCTG-3ʹ
Probe-UPL #52
Total hTERT (exon15/16)
5ʹ-GGGTCACTCAGGACAGCCCAG-3ʹ
5ʹ-GGGCGGGTGGCCATCAGT-3ʹ
Probe-UPL #37
Minus beta hTERT (exon6/9)
5ʹ-CAAGAGCCACGTCCTACGTC-3ʹ
5ʹ-CAAGAAATCATCCACCAAACG-3ʹ
Probe-UPL #58
3ʹUTR of endogenous hTERT
5ʹ-CAGCTTTTCCTCACCAGGAG-3ʹ
5ʹ-GGTCACTCCAAATTCCCAGA-3ʹ

### Bisulfite Sequencing

100 ng of gDNA was modified using the EpiTect Bisulfite kit by following the manufacturer’s instructions (Qiagen, Valencia, CA, USA). Modified DNA was PCR-amplified and cloned into the T vector system (Promega, Madison, WI, USA). 7~10 bacterial clones were sequenced for methylation analysis. Primers for the h*TERT* promoter region amplification were designed as previously described [33].

### dd3C (Droplet Digital Chromatin Conformation Capture)

Chromatin conformation capture (3C) was performed as previously described [12]. Five million cells were washed with PBS and fixed with 25 ml of medium containing 1% formaldehyde for 10 min at room temperatures. To quench the crosslinking reaction, 1.5 ml of 2.5 M glycine was added and incubated for 10 min at room temperature, followed by an additional 15 min of incubation at 4°C. Cells were washed with PBS and harvested into 1 ml of cold-PBS with protease inhibitor. Cells were next lysed by homogenization, and the nuclear pellet was collected by centrifugation. The nuclear pellet was washed and resuspended in 500 μl of ice-cold NEBuffer 2 (NEB, Ipswich, MA, USA). 15 μl of 10% SDS was added and incubated at 37°C for 1 h, followed by addition of 46.35 μl of 20% Triton X-100 for 1 h on a shaking incubator. HindIII (400U) was added and incubated overnight. Enzyme reaction was stopped by adding 88 μl of 10% SDS at 65°C for 20 min. Samples were next transferred to DNA ligation mix containing 50 mM Tris-Cl, pH 7.5, 10 mM MgCl_2_, 1 mM ATP, 10 mM DTT, and 50 μg/ml BSA. 372 μl of 20% Triton X-100 was added and incubated at 37°C for 1 h. 2,000 U of ligase (NEB, Ipswich, MA, USA) was added and incubated for 5 h at 16°C. 40 μl of 20 mg/ml Proteinase K was added to the ligation mix at 65°C overnight. DNA extraction was performed by phenol-chloroform extraction and ethanol precipitation. Quality of the libraries were determined by checking for a single DNA band under agarose gel electrophoresis. Taq-man probe and 5ʹ primers were selected to amplify constant regions at the 5p telomere regardless of genome conformation. 3ʹ primers were selected to amplify the genomic interaction between 5p telomere and subtelomeric genes up to 1.3 mega base pairs from 5p containing h*TERT*. Primer binding regions are 100 base pairs apart from a HindIII recognizing motif. Primer and probe sequences are described below;

Probe
5ʹ-[6FAM] GCCAACACAGGAATGAATTG [BHQ1]-3ʹ
Constant 5ʹ primer at 5p
5ʹ-GCCAATAAAAACAGCTACCGATG-3ʹ
3ʹ primers
5ʹ-GAGATAACTCACTACCTTCAGACCA-3ʹ (PLEKHG4B)
5ʹ-TTGAAGACATTCCTCACATCCC-3ʹ (SDHA)
5ʹ-TGTTTCCGTGATTCCTGGCAC-3ʹ (PDCD6)
5ʹ-TGGACTGTGTTGTGGGTCCTC-3ʹ (AHRR)
5ʹ-ATGCACCCACAGGTGGGTG-3ʹ (SLC9A3)
5ʹ-CTGCTGAGAAGTGTTGCCTTCT-3ʹ (CEP72)
5ʹ-ATTAGGATCACCCATCGCAG-3ʹ (TPPP)
5ʹ-TGCGCAGCATTTTGCACATG-3ʹ (ZDHHC11)
5ʹ-ATGTCGGCTTGGCCTAGAAG-3ʹ (BRD9)
5ʹ-AGGTCACTGCTGGCCTGG-3ʹ (NKD2)
5ʹ-ATGCTGGTGCCAGCTCTGAG-3ʹ (SLC12A7)
5ʹ-AGGGCTCTGGGATGTGCTG-3ʹ (SLC6A19)
5ʹ-CATTTGGAGTCCATGGAGTGAG-3ʹ (hTERT)
5ʹ-CCAGCTGTTCAGTTCAGCAGC-3ʹ (CLPTM1L)
5ʹ-TAATAGGAAGTTAACGTGCTTTGGC-3ʹ (SLC6A3)

### Chromatin Immunoprecipitation (ChIP) Analysis

Chromatin immunoprecipitation was performed as previously described [12]. Antibodies against total histone H3 and a 1:1 mixture of rabbit and mouse IgG isotypes were used as pulldown positive and negative controls of ChIP analyses, respectively. Relative occupancy was determined by first normalizing the target results with amplification signals from total H3 and then dividing by 1% input chromatin extracts. Antibodies against H3K4me3, H3K27me3, H3K9me3, H3K9ac, and LDB1 were purchased from Abcam (Cambridge, MA, USA). Antibody against TRF2 was purchased from Novus biologicals (Littleton, CO, USA). Antibody against CTCF and histone H3 was purchased from Cell signaling (Cell signaling technology, Danvers, MA, USA). Primers for h*TERT* promoter amplification were described in a previous study [33]. Primers for detection of *CLPTML1*, *SLC6A18*, *CLC6A19*, and the h*TERT*-ITS are described below;

*CLPTM1L* promoter ChIP
5ʹ- TGGGTTTGTACTGGGGAAAA
5ʹ- GAGCCTGGTGGAAGGTGATA
*SLC6A18* promoter ChIP
5ʹ- CCTGGTGTCTGCAACAAAAA
5ʹ- GCCCCACTGCAGTTGTATTT
*SLC6A19* promoter ChIP
5ʹ- TCTGGGTCCTGAACCTATGG
5ʹ- GATGTGGCCTGAATCAACCT
*TERT* ITS primer #1
5ʹ-GGAGCTGTGGTCTGTGTCTC-3ʹ
5ʹ-ACGCTAACCCTAACCCACAG-3ʹ
*TERT* ITS primer #2
5ʹ-GGGTTAGGGACACAAGCCTG-3ʹ
5ʹ-TAGAAGGGCAGGTGTCTCGT-3ʹ
*TERT* ITS primer #3
5ʹ-AGCAGACACCTGCCCTTCTA-3ʹ
5ʹ-ACTTTGTGTGCATCTGGGGA-3ʹ

### Telomere Dysfunction Induced Foci (TIF) Assay

The TIF assay is based on the co-localization detection of DNA damage by an antibody against gamma-H2AX and telomeres using FITC-conjugated telomere sequence (TTAGGG)3-specific peptide nucleic acid (PNA) probe. Briefly, BJ cells with long and short telomeres (100,000 cells) were seeded to four-well chamber slides, and, after the cells attached to the surface (next day), slides were rinsed twice with 1xPBS and fixed in 4% formaldehyde (ThermoScientific, IL) in PBS for 10 min. Then, cells were washed twice with PBS and permeabilized in 0.5% Triton X-100 in PBS for 10 min. Following permeabilization, cells were washed three times with PBS. Cells were blocked with 10% goat serum in 0.1% PBST (TritonX-100) for 1 h. Gamma-H2AX (mouse) (Millipore, Billerica, MA) was diluted 1:1,000 in blocking solution and incubated on cells for 2 h. Following three washes with PBST (1x PBS in 0.1% Triton) and three washes with PBS, cells were incubated with Alexaflour 568 conjugated goat anti mouse (1:500) (Invitrogen, Grand Island, NY) for 40 min, then washed five times with 0.1% PBST. Cells were fixed in 4% formaldehyde in PBS for 20 min at room temperature. The slides were sequentially dehydrated with 70%, 90%, and 100% ethanol. Following dehydration, denaturation was conducted with hybridization buffer containing FITC-conjugated telomere sequence (TTAGGG)_3_-specific peptide nucleic acid (PNA) probe (PNA Bio, Thousand Oaks, CA), 70% formamide, 30% 2xSSC, 10% (w/v) MgCl_2_.6*H_2_0 (Fisher Sci), 0.25% (w/v) blocking reagent for nucleic acid hybridization and detection (Roche) for 7 min at 80°C on heat block, followed by overnight incubation at room temperature. Slides were washed sequentially with 70% formamide (Ambion, Life Technologies, Grand Island, NY) / 0.6 x SSC (Invitrogen) (2 x 1 h), 2 x SSC (1 x 15 min), PBS (1 x 5 min), and sequentially dehydrated with 70%, 90%, and 100% ethanol, then mounted with Vectashield mounting medium with DAPI (Vector Laboratories, Burlingame, CA). Images were captured with Deltavision wide-field microscope using the 60X objective. TIFs were quantified using Image J and representative pictures were prepared in Imaris software after deconvolution using Autoquant X3.

### Terminal Restriction Fragmental (TRF) Length Analysis for Mean Telomere Length

The average length of telomeres (terminal restriction fragment lengths) was measured as described in [50] with the following modifications. DNA was transferred to Hybond-N+ membranes (GE Healthcare, Piscataway, NJ) using vacuum transfer. The membrane was air-dried and DNA was fixed by UV-crosslinking. Membranes were then probed for telomeres using a DIG-labeled telomere probe [51], detected with an HRP-linked anti-DIG antibody (Roche), and exposed with CDP-star (Roche).

## Supporting Information

S1 Fig Expression of intermediate genes between 5p telomere and hTERT-CLPTM1L locus. (A) Schematic map of relative location of intermediate genes are depicted. mRNA expression in BJ cells at PD34 and PD74, BJ cells with long telomeres (13 kb) and short telomeres (9kb) and IMR 90 cells at PD20 and PD51 were analyzed. RNA (1000 ng) was reverse-transcribed and diluted 1:4 prior to ddPCR and specific probes were used to assess the number of mRNA molecules per reaction. Data are presented as means and standard errors of biological replicates and technical triplicates (6 data points). Student’s paired T test determined significance. *p<0.05. (B) Western blot of CLPTM1L with total histone H3 as loading control at various population doublings in BJ fibroblasts and with expression of hTERT cDNA at different doublings post addition of hTERT. (C) RT-PCR analysis of TERT 5’UTR/exon 1 and exons 5 through 9 in young (long telomere) and old (short telomere) fibroblasts. We included human H9 stem cells as a telomerase positive control. This is a qualitative analysis only as 55 cycles of PCR were performed to detect adequate levels of hTERT transcripts in young BJ cells so we could visualize them on a gel. Quantification was performed using droplet digital PCR shown in Fig 1. Data associated with this figure can be found in the supplemental data file (S1 Data).
(TIFF)

S2 Fig Location of 3D-FISH probes against hTERT. Intermediate, and sub-telomere loci are also described on the map. Box-and-whisker plots showing single allele representation of distance between probes in 3D-FISH experiments. (**A**) Average distance between probes against h*TERT* locus and sub-telomeric region 5p was assessed in normal BJ cells at PD20 and PD90. Adjacent allele (A) and separated allele (S) were visually determined, and the distance was assessed by Imaris software. (**B**) Average distance between probes against hTERT locus and sub-telomeric region 5p was also assessed in cloned BJ cells with different telomere lengths. The proximity of allele pairs was determined visually and quantitated. (**C**) IMR90 young cell 3D FISH quantification as above with representative micrograph, scale bar = 2 microns. (**D**) IMR90 old cell 3D FISH quantification with representative micrograph, scale bar = 3 microns. (**E**) SW39 and SW26 SV40 large T antigen transformed cell 3D FISH quantification with representative micrograph, scale bar = 3 microns. (**F**) Long and short telomere BJ cells stained with telomere probe (green), nuclear DNA probe (DAPI, blue) and DNA damage (gH2A.X, red) in cells that were treated with 100 mg/mL of zeocin for 48 hrs or not (control). Scale bar = 5 microns. Data associated with this figure can be found in the supplemental data file (S1 Data).
(TIFF)

S3 Fig Difference of conformation are restricted between the hTERT and the sub-telomeric 5p. (A) Green fluorescent probe against intermediate region (RP11-846K3) between the sub-telomeric 5p and the hTERT locus was selected as a control. Red fluorescent probe stained sub-telomeric 5p region. (B) Representative deconvolved image shows no conformation change in genome structure between sub-telomeric 5p and RP11-846K3. (C) Box-and-whisker plots showing average distance between two probes assessed by Imaris software. (D) Two fluorescent probes against intermediate region on chromosome 5p (RP11-162J5: Green, RP11-5H14: Red) were selected as a control. Green and red probes are 25.5MB and 30.6MB apart from telomere respectively. (E) Representative deconvolved image shows no conformation change in genome structure between two control loci. (F) Box-and-whisker plots showing average distance between two probes assessed by Imaris software. Data associated with this figure can be found in the supplemental data file (S1 Data).
(TIFF)

S4 Fig ChIP analysis of TERT promoter. ChIP was performed as in Fig 3. Data are presented as means and standard errors of biological and technical duplicates. Student’s paired T test determine significance. *p<0.05. Data associated with this figure can be found in the supplemental data file (S1 Data).
(TIFF)

S5 Fig Validation of genome editing at chromosome 5p. (A) A Taq-man probe was designed to bind next to sgRNA target sequence. PCR amplification of flanking sequences hydrolyzes the probe to emit positive signals. (B) ddPCR amplification of 5p end region was performed with genomic DNA from Cas9-infected cells. The number of positive signals shows the approximate level of intact 5p end structure. (C) Metaphase spread analysis of Cas9-infected cells shows telomere signals at the end of chromosome 5p. 21% of chromosomes showed two telomere signals at 5p ends, while 79% of chromosomes lost at least one signal in Cas9-infected cells. Data associated with this figure can be found in the supplemental data file (S1 Data).
(TIFF)

S6 Fig Lack of telomerase activity in BJ cells with p21 knockdown. (A) BJ cells with long and short telomeres had p21 knocked-down with shRNAs. Telomerase enzyme activity (TRAP- gel based) and (B) Droplet-digital TRAP were performed and no telomerase activity was detected above background. ITAS = internal telomerase activity standard. Data associated with this figure can be found in the supplemental data file (S1 Data).
(TIFF)

S1 Data Data. (XLSX)

## Abbreviations

A: adjacent allele
ALT: alternative lengthening of telomeres
CLPTM1L: cleft lip and palate-associated transmembrane protein 1-like
CRISPR/Cas9: clustered regularly interspaced short palindromic repeat-associated 9
ITS: internal (interstitial) telomeric sequences
PD: population doubling; S, separated allele
3C: chromatin conformation capture
3D-FISH: three-dimensional fluorescence in situ hybridization
TPE-OLD: telomere position effect—over long distances
TRF: terminal restriction fragment

## References

[1] de Lange T. Shelterin: the protein complex that shapes and safeguards human telomeres. Genes Dev. 2005;19(18):2100–10. Epub 2005/09/17. 10.1101/gad.1346005 16166375

[2] Giraud-Panis MJ, Teixeira MT, Geli V, Gilson E. CST meets shelterin to keep telomeres in check. Mol Cell. 2010;39(5):665–76. 10.1016/j.molcel.2010.08.024 20832719

[3] Armanios M. Telomeres and age-related disease: how telomere biology informs clinical paradigms. The Journal of clinical investigation. 2013;123(3):996–1002. PubMed Central PMCID: PMC3673231. 10.1172/JCI66370 23454763 PMC3673231

[4] Ozturk S. Telomerase activity and telomere length in male germ cells. Biol Reprod. 2015;92(2):53 10.1095/biolreprod.114.124008 25568305

[5] Alder JK, Barkauskas CE, Limjunyawong N, Stanley SE, Kembou F, Tuder RM, et al Telomere dysfunction causes alveolar stem cell failure. Proc Natl Acad Sci U S A. 2015;112(16):5099–104. PubMed Central PMCID: PMC4413294. 10.1073/pnas.1504780112 25840590 PMC4413294

[6] Cong YS, Wright WE, Shay JW. Human telomerase and its regulation. Microbiology and molecular biology reviews: MMBR. 2002;66(3):407–25, table of contents. PubMed Central PMCID: PMC120798. 10.1128/MMBR.66.3.407-425.2002 12208997 PMC120798

[7] Hiyama E, Hiyama K. Telomere and telomerase in stem cells. Br J Cancer. 2007;96(7):1020–4. PubMed Central PMCID: PMC2360127. 10.1038/sj.bjc.6603671 17353922 PMC2360127

[8] Wright WE, Piatyszek MA, Rainey WE, Byrd W, Shay JW. Telomerase activity in human germline and embryonic tissues and cells. Dev Genet. 1996;18(2):173–9. 10.1002/(SICI)1520-6408(1996)18:2<173::AID-DVG10>3.0.CO;2-3 8934879

[9] Kim NW, Piatyszek MA, Prowse KR, Harley CB, West MD, Ho PL, et al Specific association of human telomerase activity with immortal cells and cancer. Science. 1994;266(5193):2011–5. Epub 1994/12/23. 7605428 10.1126/science.7605428

[10] Lieberman-Aiden E, van Berkum NL, Williams L, Imakaev M, Ragoczy T, Telling A, et al Comprehensive mapping of long-range interactions reveals folding principles of the human genome. Science. 2009;326(5950):289–93. PubMed Central PMCID: PMC2858594. 10.1126/science.1181369 19815776 PMC2858594

[11] Dixon JR, Selvaraj S, Yue F, Kim A, Li Y, Shen Y, et al Topological domains in mammalian genomes identified by analysis of chromatin interactions. Nature. 2012;485(7398):376–80. PubMed Central PMCID: PMC3356448. 10.1038/nature11082 22495300 PMC3356448

[12] Robin JD, Ludlow AT, Batten K, Magdinier F, Stadler G, Wagner KR, et al Telomere position effect: regulation of gene expression with progressive telomere shortening over long distances. Genes Dev. 2014;28(22):2464–76. PubMed Central PMCID: PMC4233240. 10.1101/gad.251041.114 25403178 PMC4233240

[13] Robin JD, Ludlow AT, Batten K, Gaillard MC, Stadler G, Magdinier F, et al SORBS2 transcription is activated by telomere position effect-over long distance upon telomere shortening in muscle cells from patients with facioscapulohumeral dystrophy. Genome Res. 2015. 10.1101/gr.190660.115 PMC4665000 26359233

[14] Lou Z, Wei J, Riethman H, Baur JA, Voglauer R, Shay JW, et al Telomere length regulates ISG15 expression in human cells. Aging (Albany NY). 2009;1(7):608–21. Epub 2010/02/17. 20157543 10.18632/aging.100066 PMC2806043

[15] Baur JA, Zou Y, Shay JW, Wright WE. Telomere position effect in human cells. Science. 2001;292(5524):2075–7. 10.1126/science.1062329 11408657

[16] James MA, Wen W, Wang Y, Byers LA, Heymach JV, Coombes KR, et al Functional characterization of CLPTM1L as a lung cancer risk candidate gene in the 5p15.33 locus. PLoS ONE. 2012;7(6):e36116 PubMed Central PMCID: PMCPMC3366984. 10.1371/journal.pone.0036116 22675468 PMC3366984

[17] Hrdlickova R, Nehyba J, Bose HR Jr. Alternatively spliced telomerase reverse transcriptase variants lacking telomerase activity stimulate cell proliferation. Mol Cell Biol. 2012;32(21):4283–96. PubMed Central PMCID: PMC3486134. 10.1128/MCB.00550-12 22907755 PMC3486134

[18] Arnoult N, Van Beneden A, Decottignies A. Telomere length regulates TERRA levels through increased trimethylation of telomeric H3K9 and HP1alpha. Nat Struct Mol Biol. 2012;19(9):948–56. 10.1038/nsmb.2364 22922742

[19] Balk B, Maicher A, Dees M, Klermund J, Luke-Glaser S, Bender K, et al Telomeric RNA-DNA hybrids affect telomere-length dynamics and senescence. Nat Struct Mol Biol. 2013;20(10):1199–205. 10.1038/nsmb.2662 24013207

[20] Mathon NF, Malcolm DS, Harrisingh MC, Cheng L, Lloyd AC. Lack of replicative senescence in normal rodent glia. Science. 2001;291(5505):872–5. 10.1126/science.1056782 11157166

[21] Horikawa I, Chiang YJ, Patterson T, Feigenbaum L, Leem SH, Michishita E, et al Differential cis-regulation of human versus mouse TERT gene expression in vivo: identification of a human-specific repressive element. Proceedings of the National Academy of Sciences of the United States of America. 2005;102(51):18437–42. PubMed Central PMCID: PMC1317953. 10.1073/pnas.0508964102 16344462 PMC1317953

[22] Wong MS, Chen L, Foster C, Kainthla R, Shay JW, Wright WE. Regulation of telomerase alternative splicing: a target for chemotherapy. Cell Rep. 2013;3(4):1028–35. PubMed Central PMCID: PMC3640656. 10.1016/j.celrep.2013.03.011 23562158 PMC3640656

[23] Wong MS, Shay JW, Wright WE. Regulation of human telomerase splicing by RNA:RNA pairing. Nature communications. 2014;5:3306 PubMed Central PMCID: PMC3948165. 10.1038/ncomms4306 24577044 PMC3948165

[24] Wong MS, Wright WE, Shay JW. Alternative splicing regulation of telomerase: a new paradigm? Trends Genet. 2014;30(10):430–8. 10.1016/j.tig.2014.07.006 25172021 PMC4190675

[25] Bell RJ, Rube HT, Kreig A, Mancini A, Fouse SF, Nagarajan RP, et al The transcription factor GABP selectively binds and activates the mutant TERT promoter in cancer. Science. 2015. 10.1126/science.aab0015 PMC4456397 25977370

[26] Walker EJ, Zhang C, Castelo-Branco P, Hawkins C, Wilson W, Zhukova N, et al Monoallelic expression determines oncogenic progression and outcome in benign and malignant brain tumors. Cancer Res. 2012;72(3):636–44. 10.1158/0008-5472.CAN-11-2266 22144470

[27] Jinek M, Chylinski K, Fonfara I, Hauer M, Doudna JA, Charpentier E. A programmable dual-RNA-guided DNA endonuclease in adaptive bacterial immunity. Science. 2012;337(6096):816–21. 10.1126/science.1225829 22745249 PMC6286148

[28] Srivastava M, Nambiar M, Sharma S, Karki SS, Goldsmith G, Hegde M, et al An inhibitor of nonhomologous end-joining abrogates double-strand break repair and impedes cancer progression. Cell. 2012;151(7):1474–87. 10.1016/j.cell.2012.11.054 23260137

[29] Stadler G, Rahimov F, King OD, Chen JC, Robin JD, Wagner KR, et al Telomere position effect regulates DUX4 in human facioscapulohumeral muscular dystrophy. Nat Struct Mol Biol. 2013;20(6):671–8. PubMed Central PMCID: PMC3711615. 10.1038/nsmb.2571 23644600 PMC3711615

[30] Cong YS, Wen J, Bacchetti S. The human telomerase catalytic subunit hTERT: organization of the gene and characterization of the promoter. Hum Mol Genet. 1999;8(1):137–42. 9887342 10.1093/hmg/8.1.137

[31] Bechter OE, Eisterer W, Dlaska M, Kuhr T, Thaler J. CpG island methylation of the hTERT promoter is associated with lower telomerase activity in B-cell lymphocytic leukemia. Exp Hematol. 2002;30(1):26–33. 11823034 10.1016/s0301-472x(01)00760-3

[32] Guilleret I, Yan P, Grange F, Braunschweig R, Bosman FT, Benhattar J. Hypermethylation of the human telomerase catalytic subunit (hTERT) gene correlates with telomerase activity. Int J Cancer. 2002;101(4):335–41. 10.1002/ijc.10593 12209957

[33] Zinn RL, Pruitt K, Eguchi S, Baylin SB, Herman JG. hTERT is expressed in cancer cell lines despite promoter DNA methylation by preservation of unmethylated DNA and active chromatin around the transcription start site. Cancer Res. 2007;67(1):194–201. 10.1158/0008-5472.CAN-06-3396 17210699

[34] Renaud S, Bosman FT, Benhattar J. Implication of the exon region in the regulation of the human telomerase reverse transcriptase gene promoter. Biochem Biophys Res Commun. 2003;300(1):47–54. 12480519 10.1016/s0006-291x(02)02775-4

[35] Renaud S, Loukinov D, Alberti L, Vostrov A, Kwon YW, Bosman FT, et al BORIS/CTCFL-mediated transcriptional regulation of the hTERT telomerase gene in testicular and ovarian tumor cells. Nucleic Acids Res. 2011;39(3):862–73. PubMed Central PMCID: PMC3035453. 10.1093/nar/gkq827 20876690 PMC3035453

[36] Mikkelsen TS, Ku M, Jaffe DB, Issac B, Lieberman E, Giannoukos G, et al Genome-wide maps of chromatin state in pluripotent and lineage-committed cells. Nature. 2007;448(7153):553–60. PubMed Central PMCID: PMCPMC2921165. 10.1038/nature06008 17603471 PMC2921165

[37] Shats I, Milyavsky M, Tang X, Stambolsky P, Erez N, Brosh R, et al p53-dependent down-regulation of telomerase is mediated by p21waf1. J Biol Chem. 2004;279(49):50976–85. 10.1074/jbc.M402502200 15371422

[38] Withers JB, Ashvetiya T, Beemon KL. Exclusion of exon 2 is a common mRNA splice variant of primate telomerase reverse transcriptases. PLoS ONE. 2012;7(10):e48016 PubMed Central PMCID: PMCPMC3480478. 10.1371/journal.pone.0048016 23110161 PMC3480478

[39] Wood AM, Rendtlew Danielsen JM, Lucas CA, Rice EL, Scalzo D, Shimi T, et al TRF2 and lamin A/C interact to facilitate the functional organization of chromosome ends. Nature communications. 2014;5:5467 PubMed Central PMCID: PMC4235626. 10.1038/ncomms6467 25399868 PMC4235626

[40] Simonet T, Zaragosi LE, Philippe C, Lebrigand K, Schouteden C, Augereau A, et al The human TTAGGG repeat factors 1 and 2 bind to a subset of interstitial telomeric sequences and satellite repeats. Cell Res. 2011;21(7):1028–38. PubMed Central PMCID: PMCPMC3193489. 10.1038/cr.2011.40 21423270 PMC3193489

[41] Ye J, Renault VM, Jamet K, Gilson E. Transcriptional outcome of telomere signalling. Nat Rev Genet. 2014;15(7):491–503. 10.1038/nrg3743 24913665

[42] Deng W, Lee J, Wang H, Miller J, Reik A, Gregory PD, et al Controlling long-range genomic interactions at a native locus by targeted tethering of a looping factor. Cell. 2012;149(6):1233–44. PubMed Central PMCID: PMCPMC3372860. 10.1016/j.cell.2012.03.051 22682246 PMC3372860

[43] Zhang H, Niu B, Hu JF, Ge S, Wang H, Li T, et al Interruption of intrachromosomal looping by CCCTC binding factor decoy proteins abrogates genomic imprinting of human insulin-like growth factor II. J Cell Biol. 2011;193(3):475–87. PubMed Central PMCID: PMCPMC3087012. 10.1083/jcb.201101021 21536749 PMC3087012

[44] Hastie ND, Dempster M, Dunlop MG, Thompson AM, Green DK, Allshire RC. Telomere reduction in human colorectal carcinoma and with ageing. Nature. 1990;346(6287):866–8. 10.1038/346866a0 2392154

[45] Jia W, Wang S, Horner JW, Wang N, Wang H, Gunther EJ, et al A BAC transgenic reporter recapitulates in vivo regulation of human telomerase reverse transcriptase in development and tumorigenesis. FASEB J. 2011;25(3):979–89. PubMed Central PMCID: PMC3042838. 10.1096/fj.10-173989 21135040 PMC3042838

[46] Zhu J, Zhao Y, Wang S. Chromatin and epigenetic regulation of the telomerase reverse transcriptase gene. Protein Cell. 2010;1(1):22–32. PubMed Central PMCID: PMCPMC3683535. 10.1007/s13238-010-0014-1 21203995 PMC3683535

[47] Zhao Y, Wang S, Popova EY, Grigoryev SA, Zhu J. Rearrangement of upstream sequences of the hTERT gene during cellular immortalization. Genes Chromosomes Cancer. 2009;48(11):963–74. PubMed Central PMCID: PMC2889911. 10.1002/gcc.20698 19672873 PMC2889911

[48] Peifer M, Hertwig F, Roels F, Dreidax D, Gartlgruber M, Menon R, et al Telomerase activation by genomic rearrangements in high-risk neuroblastoma. Nature. 2015;526(7575):700–4. 10.1038/nature14980 26466568 PMC4881306

[49] Ludlow AT, Robin JD, Sayed M, Litterst CM, Shelton DN, Shay JW, et al Quantitative telomerase enzyme activity determination using droplet digital PCR with single cell resolution. Nucleic Acids Res. 2014;42(13):e104 PubMed Central PMCID: PMC4117742. 10.1093/nar/gku439 24861623 PMC4117742

[50] Zhao Y, Sfeir AJ, Zou Y, Buseman CM, Chow TT, Shay JW, et al Telomere extension occurs at most chromosome ends and is uncoupled from fill-in in human cancer cells. Cell. 2009;138(3):463–75. PubMed Central PMCID: PMCPMC2726829. 10.1016/j.cell.2009.05.026 19665970 PMC2726829

[51] Lai TP, Wright WE, Shay JW. Generation of digoxigenin-incorporated probes to enhance DNA detection sensitivity. Biotechniques. 2016;60(6):306–9. 10.2144/000114427 27286808

