# Supplementary figures and images for: Regulation of the Human Telomerase Gene TERT by Telomere Position Effect—Over Long Distances (TPE-OLD): Implications for Aging and Cancer
Source: PLoS Biol. 2016 Dec 15;14(12):e2000016. doi: 10.1371/journal.pbio.2000016 (PMC5169358; doi:10.1371/journal.pbio.2000016)

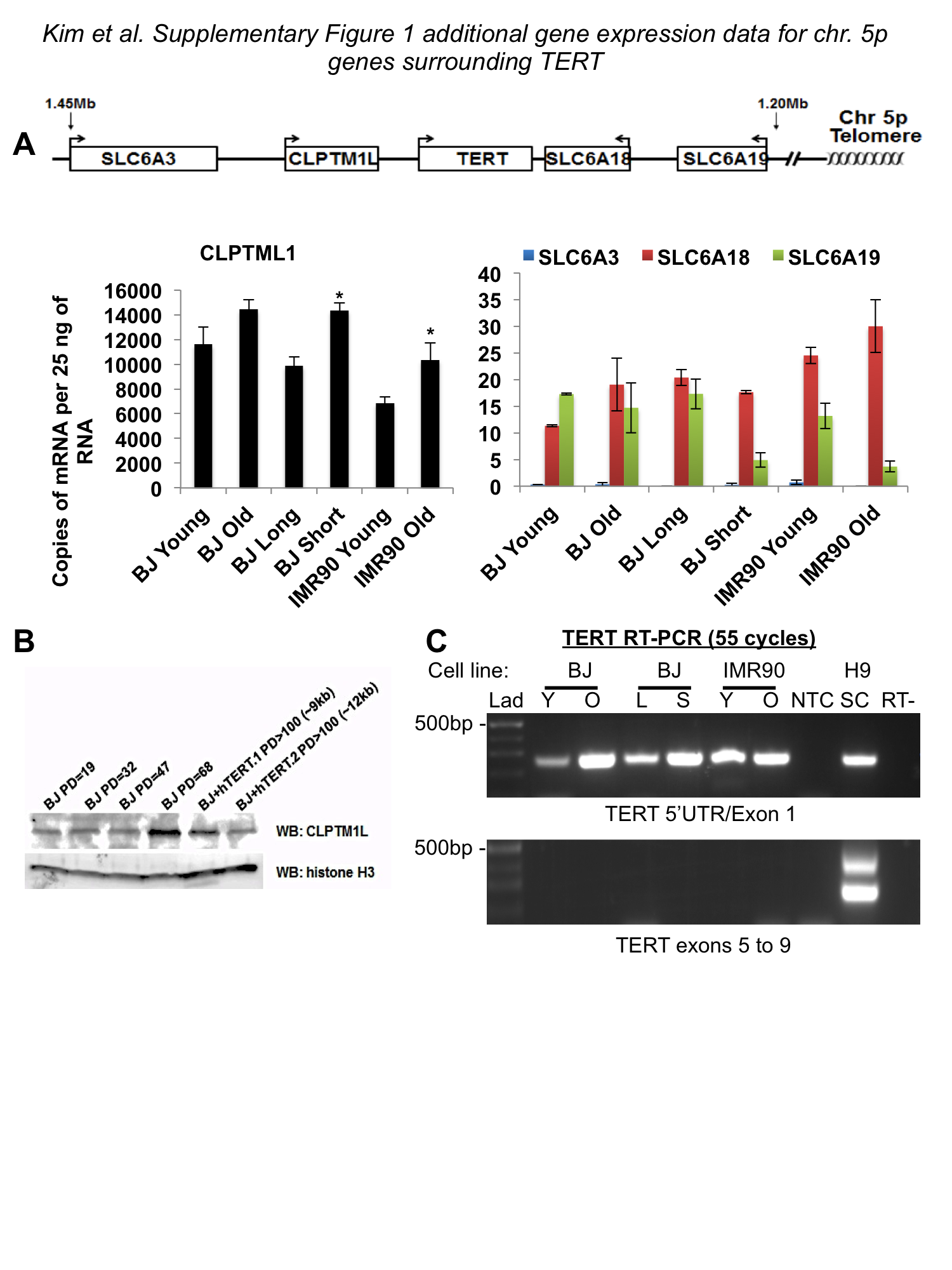

Supplement: S1 Fig — (A) Schematic map of relative location of intermediate genes are depicted. mRNA expression in BJ cells at PD34 and PD74, BJ cells with long telomeres (13 kb) and short telomeres (9kb) and IMR 90 cells at PD20 and PD51 were analyzed. RNA (1000 ng) was reverse-transcribed and diluted 1:4 prior to ddPCR and specific probes were used to assess the number of mRNA molecules per reaction. Data are presented as means and standard errors of biological replicates and technical triplicates (6 data points). Student’s paired T test determined significance. *p<0.05. (B) Western blot of CLPTM1L with total histone H3 as loading control at various population doublings in BJ fibroblasts and with expression of hTERT cDNA at different doublings post addition of hTERT. (C) RT-PCR analysis of TERT 5’UTR/exon 1 and exons 5 through 9 in young (long telomere) and old (short telomere) fibroblasts. We included human H9 stem cells as a telomerase positive control. This is a qualitative analysis only as 55 cycles of PCR were performed to detect adequate levels of hTERT transcripts in young BJ cells so we could visualize them on a gel. Quantification was performed using droplet digital PCR shown in Fig 1. Data associated with this figure can be found in the supplemental data file (S1 Data). (TIFF) [file pbio.2000016.s001.tiff]

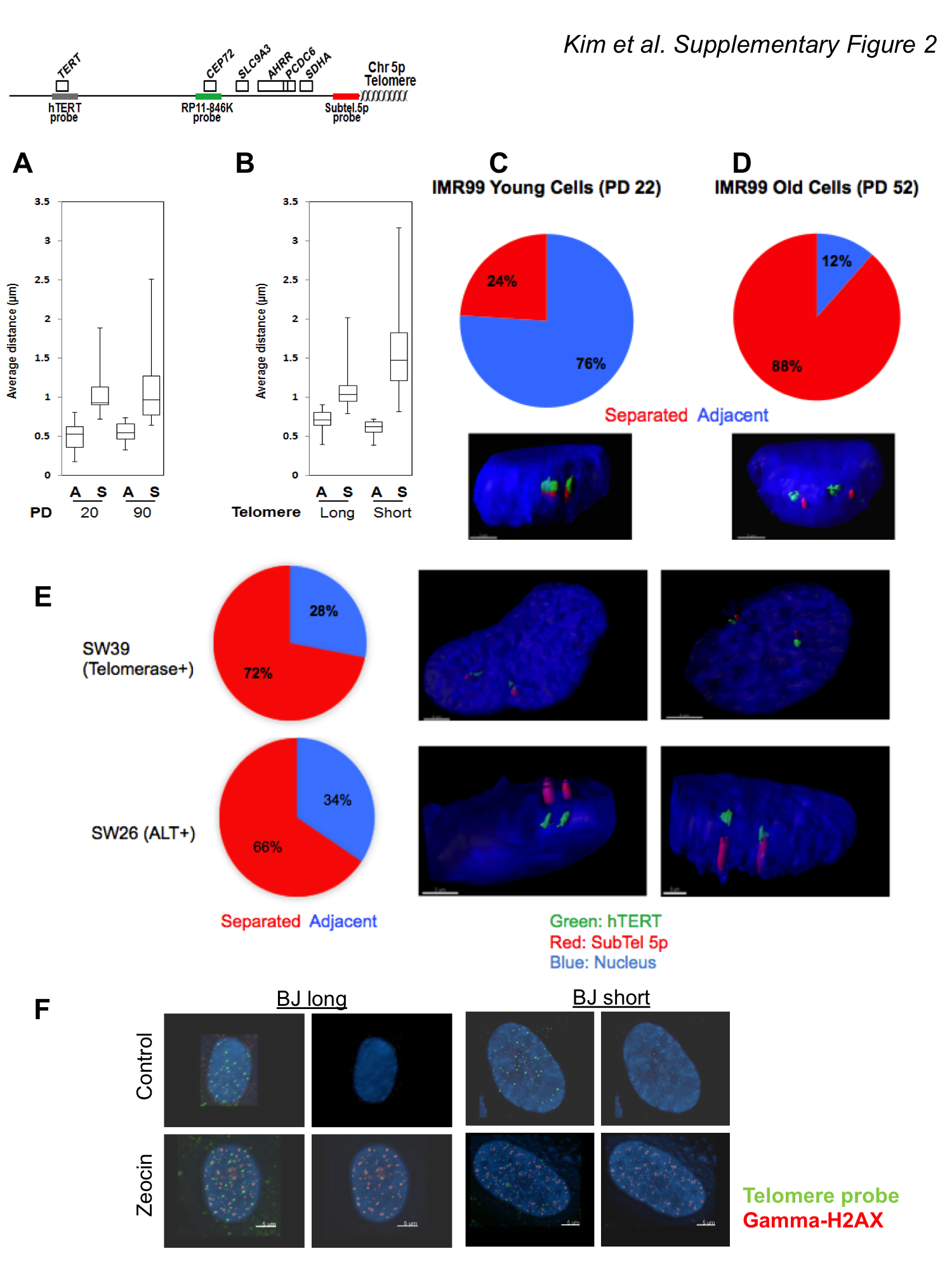

Supplement: S2 Fig — Intermediate, and sub-telomere loci are also described on the map. Box-and-whisker plots showing single allele representation of distance between probes in 3D-FISH experiments. (A) Average distance between probes against hTERT locus and sub-telomeric region 5p was assessed in normal BJ cells at PD20 and PD90. Adjacent allele (A) and separated allele (S) were visually determined, and the distance was assessed by Imaris software. (B) Average distance between probes against hTERT locus and sub-telomeric region 5p was also assessed in cloned BJ cells with different telomere lengths. The proximity of allele pairs was determined visually and quantitated. (C) IMR90 young cell 3D FISH quantification as above with representative micrograph, scale bar = 2 microns. (D) IMR90 old cell 3D FISH quantification with representative micrograph, scale bar = 3 microns. (E) SW39 and SW26 SV40 large T antigen transformed cell 3D FISH quantification with representative micrograph, scale bar = 3 microns. (F) Long and short telomere BJ cells stained with telomere probe (green), nuclear DNA probe (DAPI, blue) and DNA damage (gH2A.X, red) in cells that were treated with 100 mg/mL of zeocin for 48 hrs or not (control). Scale bar = 5 microns. Data associated with this figure can be found in the supplemental data file (S1 Data). (TIFF) [file pbio.2000016.s002.tiff]

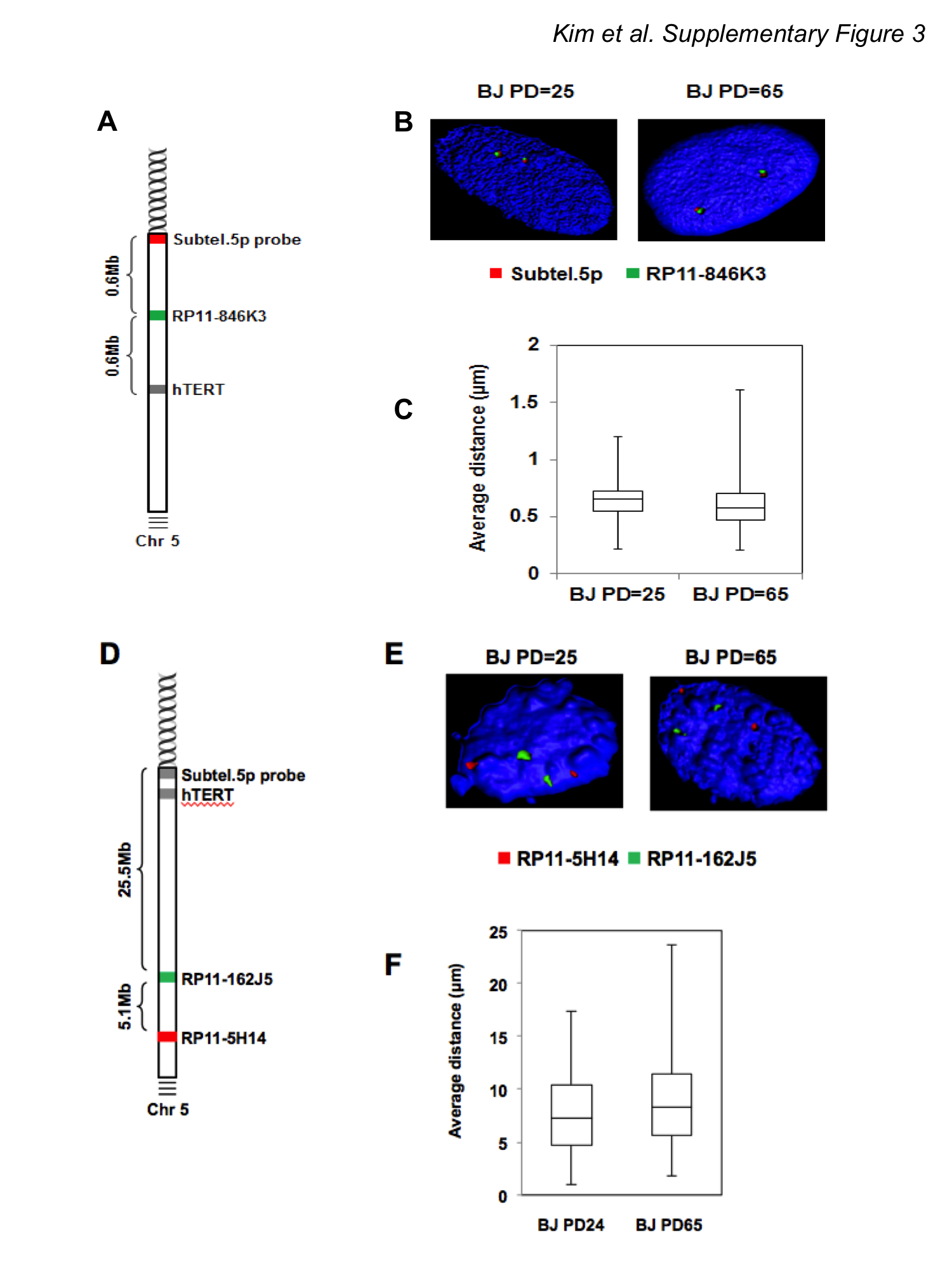

Supplement: S3 Fig — (A) Green fluorescent probe against intermediate region (RP11-846K3) between the sub-telomeric 5p and the hTERT locus was selected as a control. Red fluorescent probe stained sub-telomeric 5p region. (B) Representative deconvolved image shows no conformation change in genome structure between sub-telomeric 5p and RP11-846K3. (C) Box-and-whisker plots showing average distance between two probes assessed by Imaris software. (D) Two fluorescent probes against intermediate region on chromosome 5p (RP11-162J5: Green, RP11-5H14: Red) were selected as a control. Green and red probes are 25.5MB and 30.6MB apart from telomere respectively. (E) Representative deconvolved image shows no conformation change in genome structure between two control loci. (F) Box-and-whisker plots showing average distance between two probes assessed by Imaris software. Data associated with this figure can be found in the supplemental data file (S1 Data). (TIFF) [file pbio.2000016.s003.tiff]

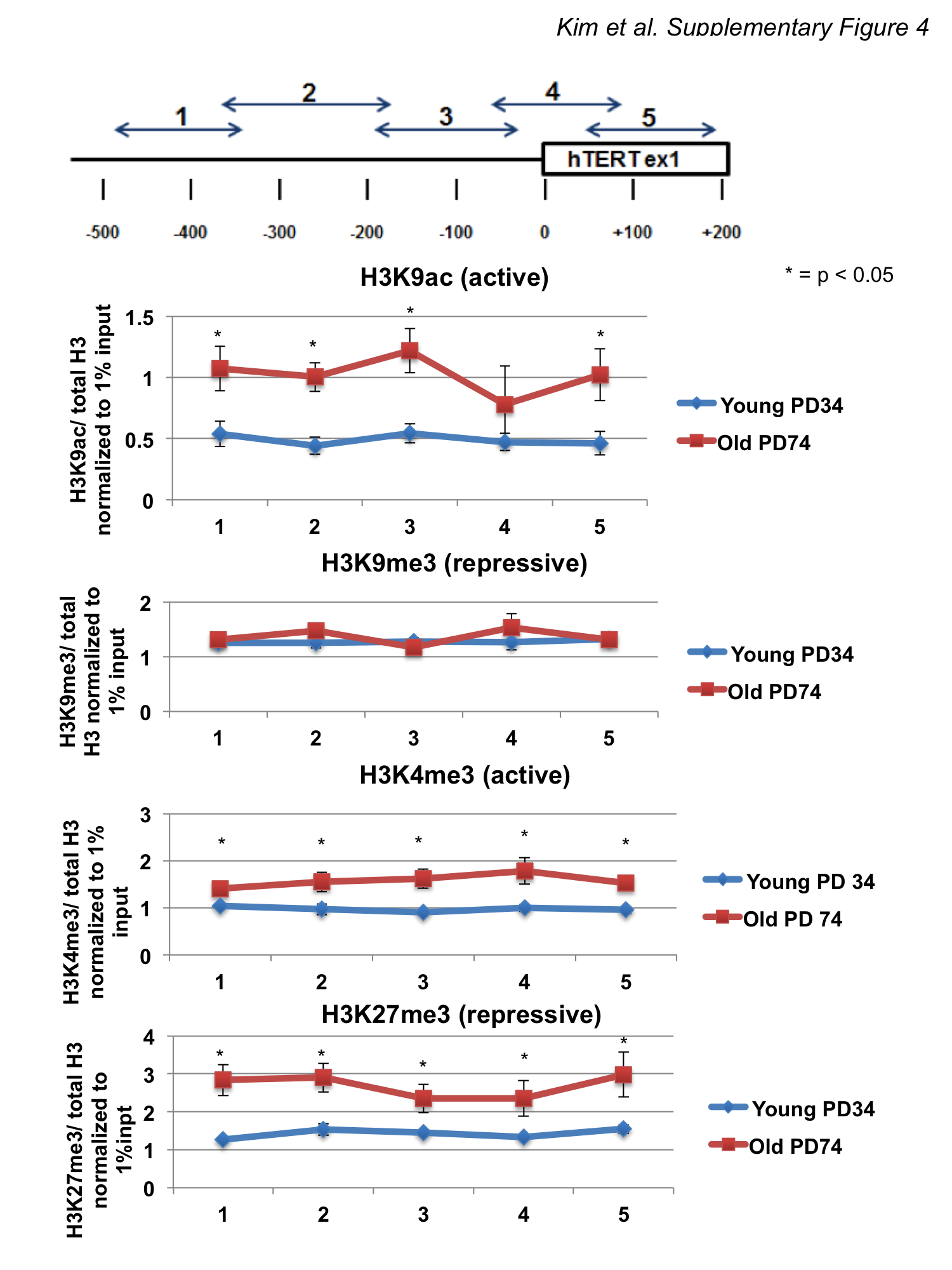

Supplement: S4 Fig — ChIP was performed as in Fig 3. Data are presented as means and standard errors of biological and technical duplicates. Student’s paired T test determine significance. *p<0.05. Data associated with this figure can be found in the supplemental data file (S1 Data). (TIFF) [file pbio.2000016.s004.tiff]

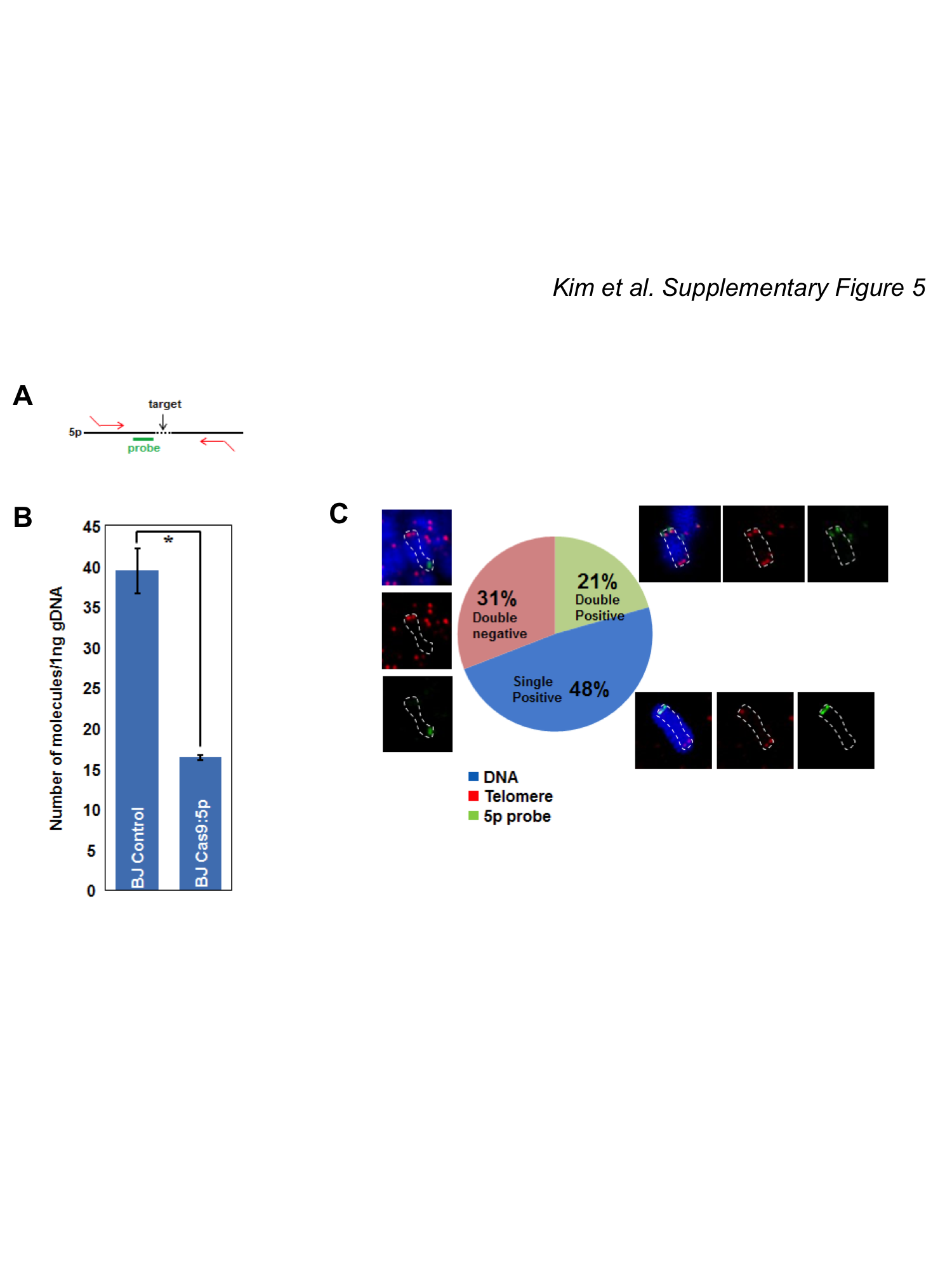

Supplement: S5 Fig — (A) A Taq-man probe was designed to bind next to sgRNA target sequence. PCR amplification of flanking sequences hydrolyzes the probe to emit positive signals. (B) ddPCR amplification of 5p end region was performed with genomic DNA from Cas9-infected cells. The number of positive signals shows the approximate level of intact 5p end structure. (C) Metaphase spread analysis of Cas9-infected cells shows telomere signals at the end of chromosome 5p. 21% of chromosomes showed two telomere signals at 5p ends, while 79% of chromosomes lost at least one signal in Cas9-infected cells. Data associated with this figure can be found in the supplemental data file (S1 Data). (TIFF) [file pbio.2000016.s005.tiff]

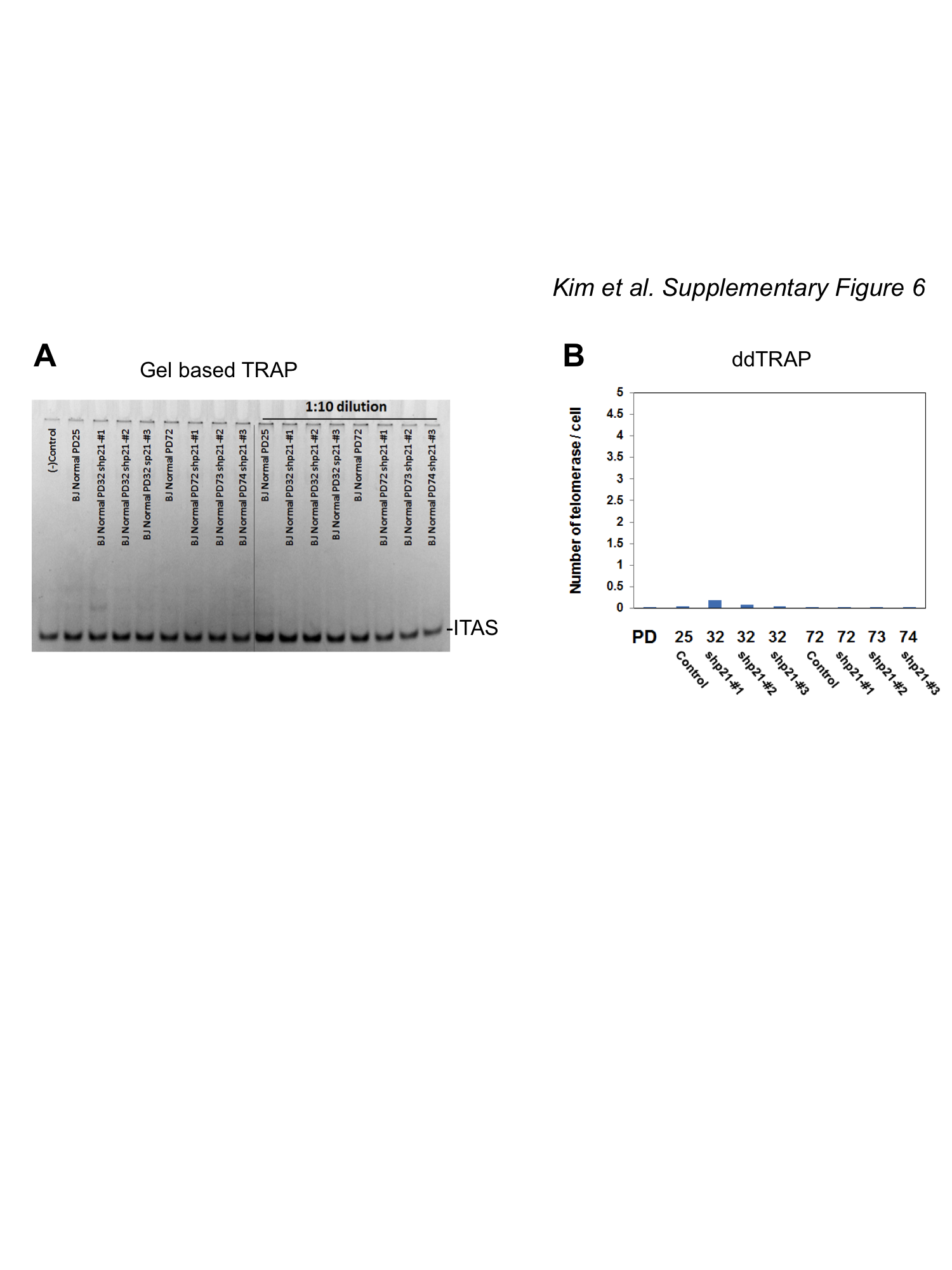

Supplement: S6 Fig — (A) BJ cells with long and short telomeres had p21 knocked-down with shRNAs. Telomerase enzyme activity (TRAP- gel based) and (B) Droplet-digital TRAP were performed and no telomerase activity was detected above background. ITAS = internal telomerase activity standard. Data associated with this figure can be found in the supplemental data file (S1 Data). (TIFF) [file pbio.2000016.s006.tiff]
